# Identification of tissue‐specific transcriptional markers of caloric restriction in the mouse and their use to evaluate caloric restriction mimetics

**DOI:** 10.1111/acel.12608

**Published:** 2017-05-26

**Authors:** Jamie L. Barger, James M. Vann, Nicole L. Cray, Thomas D. Pugh, Angela Mastaloudis, Shelly N. Hester, Steven M. Wood, Michael A. Newton, Richard Weindruch, Tomas A. Prolla

**Affiliations:** ^1^ LifeGen Technologies LLC Madison WI USA; ^2^ Center for Anti‐Aging Research NSE Products, Inc. Provo UT USA; ^3^ Departments of Statistics and of Biostatistics and Medical Informatics University of Wisconsin Madison WI USA; ^4^ Department of Medicine SMPH University of Wisconsin Madison WI USA; ^5^ Geriatric Research, Education and Clinical Center William S. Middleton Memorial Veterans Hospital Madison WI USA; ^6^ Departments of Genetics and Medical Genetics University of Wisconsin Madison WI USA

**Keywords:** aging, biomarkers, caloric restriction, gene expression, mouse

## Abstract

Caloric restriction (CR) without malnutrition has been shown to retard several aspects of the aging process and to extend lifespan in different species. There is strong interest in the identification of CR mimetics (CRMs), compounds that mimic the beneficial effects of CR on lifespan and healthspan without restriction of energy intake. Identification of CRMs in mammals is currently inefficient due to the lack of screening tools. We have performed whole‐genome transcriptional profiling of CR in seven mouse strains (C3H/HeJ, CBA/J, DBA/2J, B6C3F1/J, 129S1/SvImJ, C57BL/6J, and BALB/cJ) in white adipose tissue (WAT), gastrocnemius muscle, heart, and brain neocortex. This analysis has identified tissue‐specific panels of genes that change in expression in multiple mouse strains with CR. We validated a subset of genes with qPCR and used these to evaluate the potential CRMs bezafibrate, pioglitazone, metformin, resveratrol, quercetin, 2,4‐dinitrophenol, and L‐carnitine when fed to C57BL/6J 2‐month‐old mice for 3 months. Compounds were also evaluated for their ability to modulate previously characterized biomarkers of CR, including mitochondrial enzymes citrate synthase and SIRT3, plasma inflammatory cytokines TNF‐α and IFN‐γ, glycated hemoglobin (HbA1c) levels and adipocyte size. Pioglitazone, a PPAR‐γ agonist, and L‐carnitine, an amino acid involved in lipid metabolism, displayed the strongest effects on both the novel transcriptional markers of CR and the additional CR biomarkers tested. Our findings provide panels of tissue‐specific transcriptional markers of CR that can be used to identify novel CRMs, and also represent the first comparative molecular analysis of several potential CRMs in multiple tissues in mammals.

## Introduction

CR without malnutrition of micronutrients has been known for decades to profoundly increase lifespan and healthspan in multiple strains of laboratory rodents (Weindruch & Walford, 1988). More recent studies in model organisms (Taormina & Mirisola, 2014) and nonhuman primates (Mattison *et al*., 2012; Colman *et al*., 2014) also provide support for increased survival or prevention of age‐related pathology in these widely diverse animal models. Accordingly, there is strong interest in translating these findings to human aging. However, several recent studies suggest that the effects of CR on aging are complex. Different mouse strains respond differently to CR, ranging from life extension to life shortening (Liao *et al*., 2010). The positive effect of CR on lifespan in mouse strains appears to be correlated with maintaining the level of adiposity under CR, and efforts to identify genes that mediate these effects are ongoing (Liao *et al*., 2011). There is also interest in understanding if CR and genetic interventions that increase survival actually reduce rates of aging at the molecular level. Gene expression profiling studies of multiple tissues in aging mice have shown that CR initiated in early to mid‐life delays age‐related gene expression changes, suggesting a delay in aging at the molecular level (Park *et al*., 2009). A recent study based on the analysis of age‐dependent mortality rates suggests that CR delays aging at early ages, but is associated with a pattern that resembles a compression of the aging process in the late component of the lifespan (Koopman *et al*., 2016). Thus, the mechanisms of action of CR remain unclear, and understanding how different tissues and strains of mice respond to this dietary intervention is likely to be useful in understanding how CR impacts the aging process.

Two major, interrelated CR research directions are the following: (i) the identification of mechanisms which underlie CR's favorable health outcomes and (ii) the discovery of agents which may mimic at least some of the desirable outcomes of CR in subjects fed a normal caloric intake. The development of CR mimetics (CRMs) is important because the widespread practice of CR itself is unlikely to be practical in humans. An early consideration of how to approach the identification of CRMs focused on metabolic interventions (Weindruch *et al*., 2001). This metabolic theme proved to be a productive avenue for the discovery of CRMs based on a recent review of the subject (Roth & Ingram, 2015).

CRMs, in large part, are either drugs or phytochemicals. Drugs tested in long‐term studies for their ability to mimic aspects of CR in mice are the glycolytic inhibitor 2‐deoxy‐D‐glucose (Minor *et al*., 2010), the antidiabetic drug metformin (Martin‐Montalvo *et al*., 2013), and the mitochondrial uncoupler 2,4‐dinitrophenol (Caldeira da Silva *et al*., 2008). Both metformin and 2,4‐dinitrophenol studies reported favorable effects of these treatments on longevity and other consequences of aging, yet 2‐deoxy‐D‐glucose displayed cardiac toxicity and early mortality despite mimicking some of the metabolic effects of CR. Regarding phytochemical compounds, the most widely studied compound shown to mimic CR is resveratrol. Interestingly, high‐dose resveratrol does not appear to extend longevity of lean (genetically normal) mice (Pearson *et al*., 2008; Miller *et al*., 2011). However, we reported that mice from a long‐lived strain treated from 14 to 30 months of age with either a relatively low dose of resveratrol or CR showed fewer signs of cardiac aging than age‐matched controls (Barger *et al*., 2008b), implying positive effects on healthspan. Furthermore, there was striking mimicry of CR‐induced transcriptional shifts by resveratrol in heart, muscle, and brain in old animals. These conflicting observations suggest that CRMs may have tissue‐specific effects in aging and that a tissue‐specific screening strategy may be useful in evaluating CRMs.

In this study, we utilized a gene expression profiling approach to identify robust tissue‐specific transcriptional markers of CR that were significantly altered in expression in the majority of mouse strains tested. We focused on heart, gastrocnemius, white adipose tissue (WAT), and brain neocortex. Using quantitative PCR, we then screened seven candidate CRMs for their ability to influence the expression of some of the novel CR transcriptional markers *in vivo*. We also measured the effects of the candidate CRMs on previously characterized, nontranscriptional CR biomarkers. Our study identifies multiple transcriptional markers of CR in several tissues and provides the first comparative analysis of several putative CRMs on different tissues in mammals.

## Results

### Identification of gene expression biomarkers of CR in multiple mouse strains

We placed 2‐month‐old mice of seven mouse strains (C3H/HeJ, CBA/J, DBA/2J, B6C3F1/J, 129S1/SvImJ, C57BL/6J, and BALB/cJ) under either a CR regimen or control diet feeding for 3 months (Experimental Procedures) and performed whole‐genome gene expression profiling of selected tissues (WAT, gastrocnemius muscle, heart, and neocortex), measuring 448 distinct gene expression profiles in total. While CR effects on transcription varied by tissue and by strain, statistical analysis identified many genes with consistent across‐strain CR effects in each tissue. For each strain, we identified genes displaying an uncorrected Student's *t*‐test *P*‐value < 0.01 when comparing CR vs. control diet, independent of the magnitude or direction of the fold change. We then considered CR to have significantly altered a gene's expression if that gene populated sufficiently many of these primary, strain‐specific lists. There were only 25 genes commonly changed by CR in at least 5/7 strains in heart, and 87 genes commonly changed by CR across at least 4/7 strains in heart (Table S1, Supporting information). There were more common changes in gastrocnemius muscle as compared to heart, 143 genes changed in expression in at least 5/7 strains (Table S2, Supporting information). Common changes among strains were most abundant in WAT, with 413 genes changing in expression in at least 6/7 strains (Table S3, Supporting information). In cerebral neocortex, there were no genes that changed in expression in five or more strains, and there were only six and 22 genes changed in expression in 4/7 and 3/7 strains, respectively (Table S4, Supporting information). These multistrain, CR‐associated genes exhibited relatively high expression compared to the full transcriptome (Data S1: Fig. SS‐1, Supporting information), and the direction of significant CR effects (up vs. down) was consistent over strains (Data S1: Table SS‐4, Supporting information).

Transcriptome‐wide error‐rate control was achieved by requiring a gene to be on sufficiently many strain‐specific lists, even though *P*‐values defining the primary strain‐specific gene lists were not corrected for multiple comparison. We determined the number of genes that would be expected to show extreme *t*‐statistics in multiple strains by chance alone, that is, if the effects of CR in different mouse strains are in fact unrelated [Data S1: Fig. SS2, Supporting information]. Under this strain‐independence hypothesis, the expected values of the number of identified genes were very low (0.9, 0.3, 0.8, and three genes for heart, gastrocnemius, adipose tissue, and cerebral neocortex for the 4/7, 5/7, 6/7, and 3/7 strain possibilities mentioned above for each tissue). As an alternative approach, we combined data from all strains and used two‐way analysis of variance (ANOVA) to obtain a gene‐specific false discovery rate of an across‐strain CR effect. All genes identified by the multistrain *t*‐test rule were also highly significant by the ANOVA rule, for heart, gastrocnemius, and adipose tissue, respectively [Data S1: Fig. SS3, Supporting information]. Only one gene of the 22 genes from the 3/7 strain selection in cerebral neocortex was not highly significant based on this approach (Data S1: Fig. SS3, Supporting information). Across tissues, excluding cerebral neocortex, the genes identified as having across‐strain CR effects showed strong differential expression signatures in control vs. CR mice [Data S1: Figs SS5–SS8, Supporting information].

A pattern of tissue‐specific response to CR in WAT, gastrocnemius muscle, and heart was observed across strains, wherein CR induced the greatest overall number of changes in gene expression in WAT, followed by gastrocnemius muscle, with heart showing generally the fewest changes in expression (Table S5, Supporting information; Data S1: Figs SS‐3 and SS‐4, Supporting information). The BALB/cJ strain tended to show the fewest changes in expression overall, which reflected the observation that this is the only strain that did not show a significant reduction in body weight in response to CR (Fig. S1, Supporting information).

### Identification of common gene sets affected by CR in heart, gastrocnemius, adipose tissue, and cerebral neocortex of multiple mouse strains

**Figure 1:**
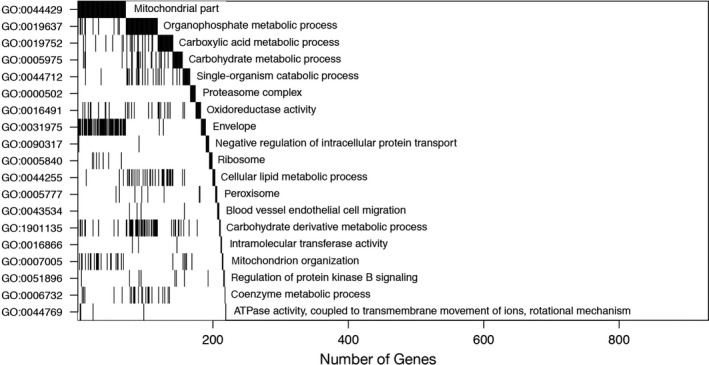
Graphical summary of gene‐set enrichment analysis of genes altered in expression in WAT in response to CR. The ‘waterfall plot’ was constructed by finding the significant (Bonferroni‐corrected *P*‐value < 0.05, Z score larger than 4.34) GO term having the largest overlap with the list of genes significantly changed with CR in multiple strains (mitochondrial part GO:0044429, 71 genes) and placing it in the top row of the figure. We next removed these genes from the list and found the significant GO term having the highest overlap with the remainder of the list (organophosphate metabolic process GO:0019637, 58 genes). The process was repeated as long as new significant GO gene sets described as‐yet‐undescribed genes in the list. Genes identified by this sequential process are counted along the *x*‐axis, and the overlap between the GO terms can be visually assessed. Shading under the ‘waterfall’ component of the graph indicates genes that were also annotated to previously named categories. The sequential coverage calculation identifies a dominant set of significant GO terms. For a full list of significant gene sets and associated z scores in adipose tissue, see Data S1: Table SS4 (Supporting information).

We performed gene‐set enrichment analysis (Bonferroni‐corrected *P*‐value < 0.05, Z score larger than 4.34) to identify Gene Ontology (GO) terms that were overrepresented among the multistrain CR‐associated genes (Figs 1 and S2–S4, Supporting information, and Data S1: Tables SS5–SS8, Supporting information). Figure 1 summarizes dominant functional categories in adipose tissue from among the 150 GO terms found to have significant overrepresentation. For example, the 413 CR‐associated genes in WAT (changing in expression in at least 6/7 strains) contain 71 genes encoding the GO term *mitochondrial components*. Other dominant GO terms in adipose tissue with CR are *organophosphate metabolic processes*,* carboxylic acid metabolic processes*, and *carbohydrate metabolic processes*. All of these categories show overlap with the *mitochondrial part* GO term, as shown in Fig. 1. Dominant GO terms in the heart (Fig. S2, Supporting information) were *single‐organism catabolic processes*,* GTP binding, mitochondrial part*, and *transition metal ion transport*. In gastrocnemius muscle (Fig. S3, Supporting information), dominant GO terms were *lipid metabolic process*,* protein oligomerization*,* I‐band*, and *cellular response to insulin stimulus*. Complete listings of GO terms can be found in the Data S1: Tables S8–S11 (Supporting information). Although there are clearly tissue‐specific responses to CR, induction of gene sets involved in mitochondrial function and energy metabolism are major components of the CR transcriptional response in these three tissues, as previously noted by us (Barger *et al*., 2015) and others (Plank *et al*., 2012) in studies analyzing multiple CR DNA microarray datasets.

### Quantitative RT–PCR analysis of a subset of genes

We selected 7, 10, and 11 transcriptional markers of CR in heart, gastrocnemius muscle, and WAT, respectively, for validation by qPCR (selection criteria described in Experimental Procedures). The effect of CR on these genes was highly consistent across three biological replicate samples as well as three technical replicates (Table S6, Supporting information). These panels of tissue‐specific CR transcriptional markers were then used in the screen of CR mimetics. Genes identified in neocortex were not analyzed by qPCR as there were too few genes that were consistently changed in expression by CR in multiple strains.

### Screening putative CRMs

For evaluation of CRMs, C57BL/6J mice were fed the putative CRM compounds (bezafibrate, resveratrol, 2‐4, DNP, quercetin, pioglitazone, metformin, L‐carnitine, dosages listed in Experimental Procedures) for 3 months, starting at 2 months of age. Of the seven transcriptional markers of CR identified and validated by qPCR in heart, neither bezafibrate, metformin, quercetin, nor resveratrol significantly modulated any of these genes (Table 1). One gene (*Scd4*) was significantly increased in expression by 2–4 dinitrophenol (1.70‐fold) and by pioglitazone (1.64‐fold); however, this change was in the opposite direction as observed with CR (−5.53‐fold). Only L‐carnitine significantly modulated three of the biomarkers in the same direction as CR in the heart (*Tfrc*,* Tuba8*, and *Chrna2*).

**Table 1:**
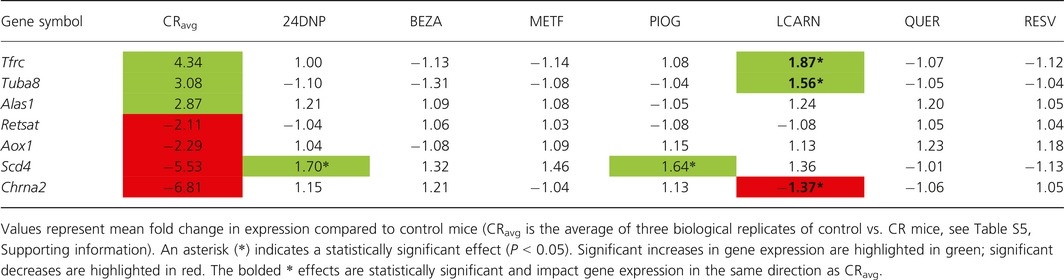
Ability of compounds to mimic the effects of CR on gene expression in a panel of seven transcriptional markers of CR in the heart evaluated by qPCR

For the 10 transcriptional markers of CR validated by qPCR in gastrocnemius muscle, pioglitazone, quercetin, L‐carnitine, and 2,4‐dinitrophenol had the greatest activity, modulating six, three, four, and three genes, respectively, in the same direction as CR (Table 2). However, pioglitazone, metformin, and quercetin, all modulated three genes in the opposite direction of CR; 2,4‐dinitrophenol, bezafibrate, and resveratrol each modulated two genes in the opposite direction of CR. Only L‐carnitine influenced genes consistently in the same direction as CR. For the 11 transcriptional markers of CR in WAT validated by qPCR, pioglitazone, L‐carnitine, quercetin, and 2,4‐dinitrophenol were the most active, modulating eight, seven, and six genes, respectively, in a manner consistent with CR. Pioglitazone and quercetin modulated two and one genes, respectively, in the opposite direction of CR. Bezafibrate, metformin, and resveratrol each modulated four genes in a manner consistent with CR, but no genes in the opposite direction of CR (Table 3). Based on this analysis, pioglitazone and L‐carnitine were the most active CR mimetics tested, altering the expression of 14 genes in total in the three tissues tested in a manner consistent with CR. Notably, pioglitazone modulated a total of six genes in the opposite direction of CR, whereas L‐carnitine did not modulate any genes in a manner inconsistent with CR mimicry.

**Table 2:**
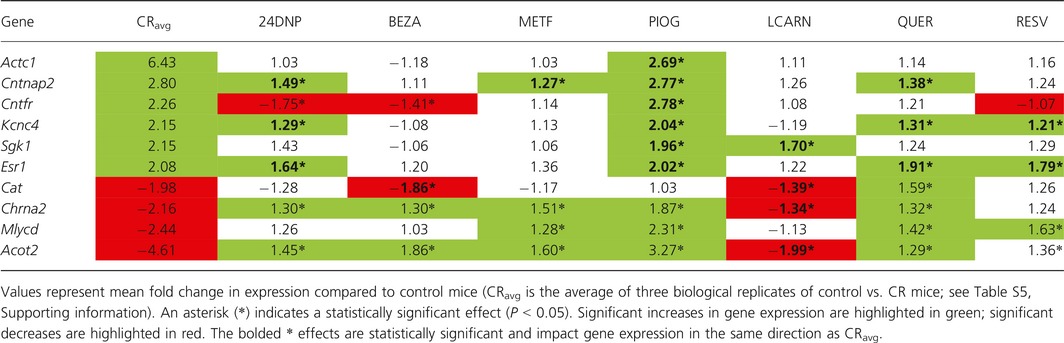
Ability of compounds to mimic the effects of CR on gene expression in a panel of 10 transcriptional markers of CR in the gastrocnemius muscle evaluated by qPCR

**Table 3:**
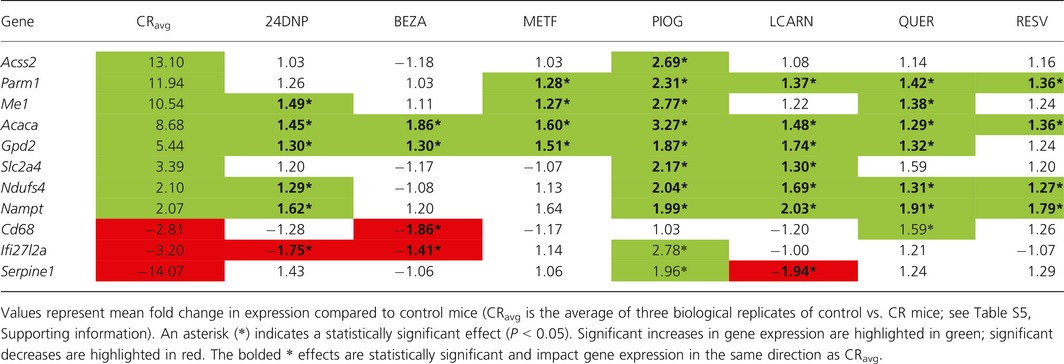
Ability of compounds to mimic the effects of CR on gene expression in a panel of 11 transcriptional markers of CR in epididymal WAT evaluated by qPCR

### CR mimicry in previously reported CR biomarkers

**Figure 2:**
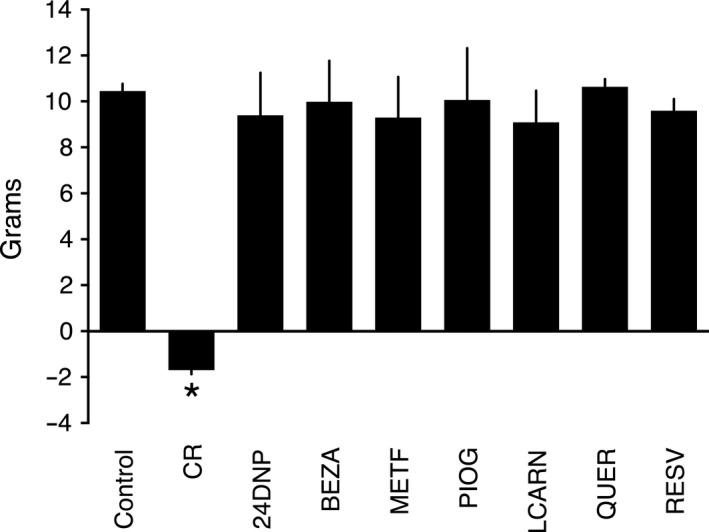
Change in body weight (22 vs. 8 weeks of age) in response to interventions. Relative to control mice, CR was the only intervention to significantly reduce body weight. Data represent means with standard error of the mean. *P < 0.05.

**Figure 3:**
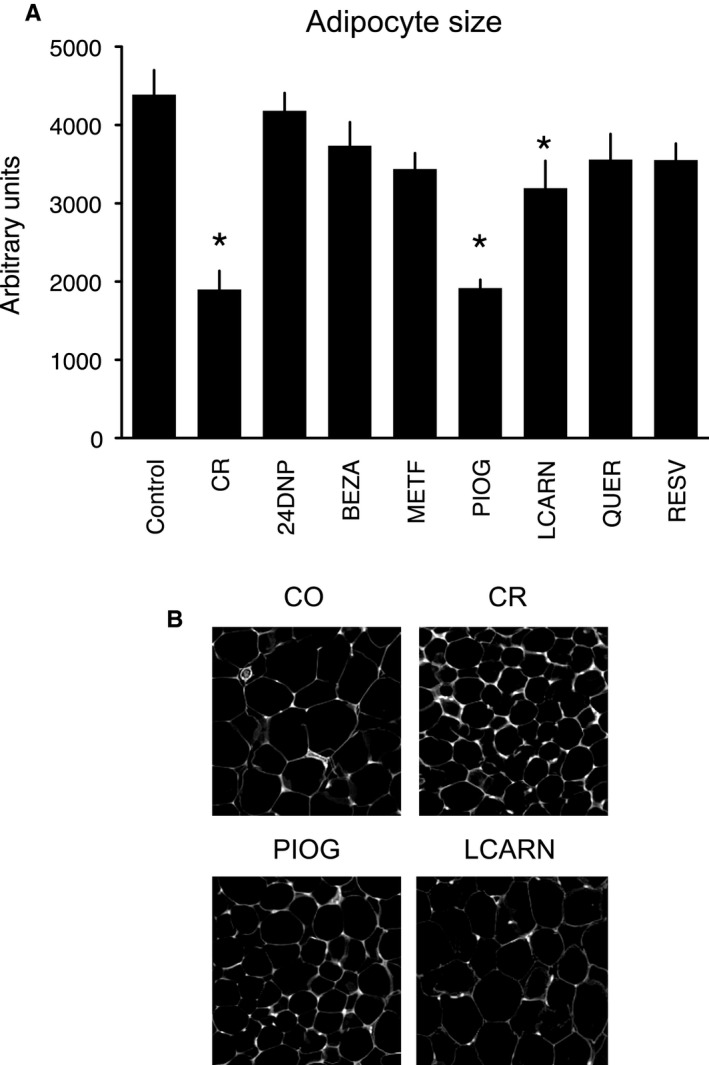
Change in adipocyte size in response to interventions. CR, PIOG, and LCARN reduced adipocyte size (A), and representative micrographs for these interventions are shown in (B). Data represent means with standard error of the mean. *P < 0.05. *n* = 5–6 per group.

**Figure 4:**
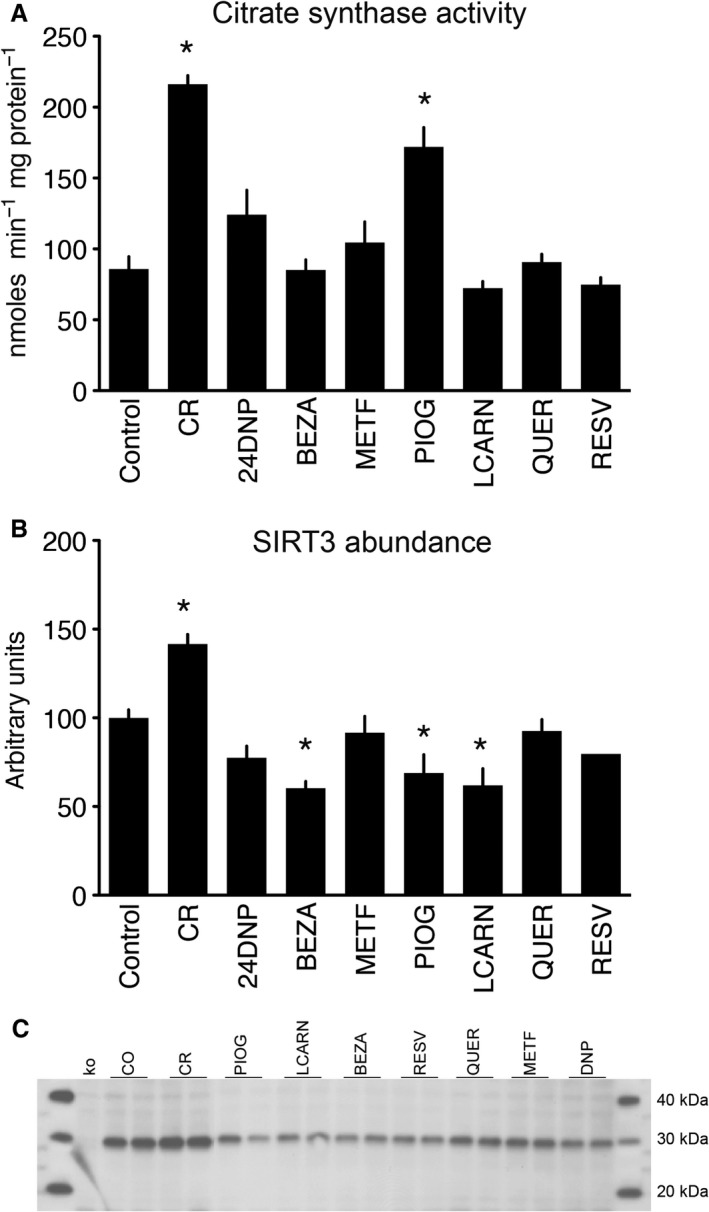
Effect of interventions on mitochondrial parameters. CR increased WAT citrate synthase activity (A) and increased the abundance of SIRT3 protein in liver (B). Only PIOG significantly mimicked the effect of CR, increasing citrate synthase activity. BEZA, PIOG, and LCARN all reduced levels of SIRT3 compared to control diet‐fed animals. Representative staining for SIRT3 (*n* = 2 samples per group) is shown in (C), along with molecular weight standards as well as a negative control from SIRT3 knockout mice (KO). Data represent means with standard error of the mean. *P < 0.05.

CR was the only intervention that induced a significant change in body weight over the course of the study (Figs 2 and S1, Supporting information). We previously reported induction of multiple energy metabolism transcripts and reduction in adipocyte size in CR mice (Higami *et al*., 2004; Barger *et al*., 2015). Alterations in adipocyte size and metabolism may be related to the previously reported increase in adiponectin and other adipocytokines in mice under CR (Wang *et al*., 2007). Adipocyte size was significantly decreased by CR, pioglitazone, and L‐carnitine (Fig. 3A,B). We observed an increase in citrate synthase (CS) activity in WAT, a marker of mitochondrial mass, in response to CR and in the pioglitazone group (Fig. 4A). CR also led to an increase in the abundance of the SIRT3 mitochondrial deacetylase in liver (Fig. 4B). However, pioglitazone, bezafibrate, and L‐carnitine all significantly decreased the abundance of SIRT3 (Fig. 4B,C).

**Figure 5:**
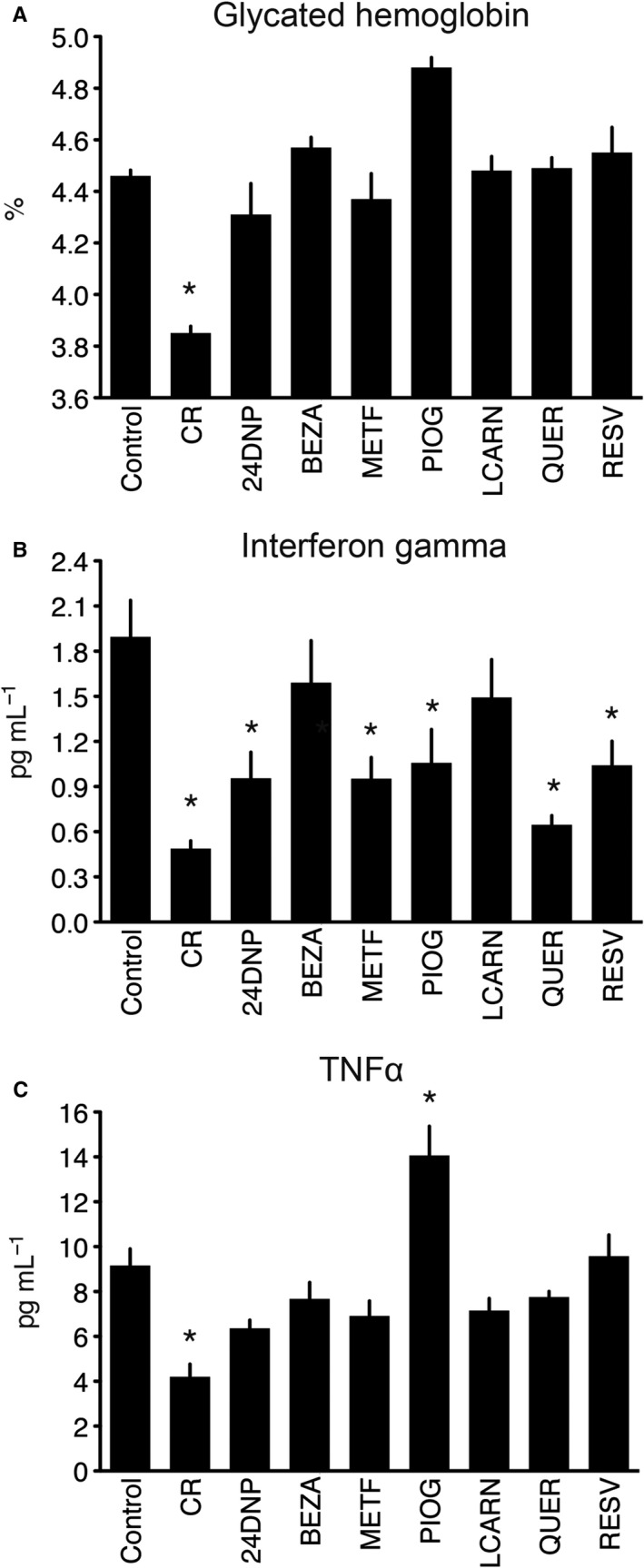
Effect of interventions on circulating biomarkers of CR. Glycated hemoglobin in whole blood (A), plasma interferon gamma (B), and plasma tumor necrosis factor α (C) were all significantly reduced by CR. Interferon gamma was also decreased by 24DNP, METF, PIOG, QUER, and RESV; however, PIOG significantly increased plasma tumor necrosis factor α. Data represent means with standard error of the mean, *n* = 10 mice per group; *P < 0.05.

Finally, we measured the levels of three circulating biomarkers of CR. Glycated hemoglobin (HbA1c) is a marker of long‐term glucose levels and its level measured in whole blood was significantly decreased only by CR (Fig. 5A). A reduction in expression of transcripts involved in inflammation is a conserved feature of delayed aging by CR (Barger *et al*., 2015), and we observed two plasma markers of inflammation (TNF‐α and IFN‐γ) were decreased in the plasma of CR mice (Fig. 5B,C). These markers were also modulated by the compounds tested, wherein 2,4‐dinitrophenol, metformin, pioglitazone, quercetin, and resveratrol all significantly lowered IFN‐γ whereas pioglitazone significantly increased TNF‐α.

## Discussion

A number of pathways have been associated with the health benefits of CR, leading to different proposals as to which pathways should be targeted by a CRM. The sirtuins, a class of NAD^+^‐dependent deacetylases, have been shown to mediate some of the effects of CR (Guarente, 2013), including lifespan extension (Satoh *et al*., 2013; Mercken *et al*., 2014; Mitchell *et al*., 2014), reduction in age‐related oxidative stress, and prevention of age‐related hearing loss (Someya *et al*., 2010). Studies in *C. elegans* suggest that induction of autophagy mediates some of the lifespan‐extending effects of CR (Morselli *et al*., 2010), and modulation of autophagy and its resulting depletion of cytosolic acetyl coenzyme A (acetyl‐CoA) have been proposed as a key target of CRMs (Mariño *et al*., 2014). One of the first suggested approaches for the design of CRMs has been modulation of energy metabolism, particularly through glycolytic inhibitors such as 2‐deoxy‐D‐glucose (Ingram & Roth, 2015). Other research suggests that mTOR inhibitors may mimic some of the effects of CR (Wilkinson *et al*., 2012), although recent studies question the relevance of these effects and the proposed shared mechanism with CR (Neff *et al*., 2013). Thus, a wide array of potential targets have been suggested to identify CRMs, and there is no universal agreement on what pathway(s) are the most relevant for CR mimicry.

Previous studies have identified genes and gene expression modules associated with CR in multiple mouse tissues, strains, and different species (Swindell, 2009; Plank *et al*., 2012; Collino *et al*., 2013; Barger *et al*., 2015). These studies have identified shared pathways activated or suppressed by CR, including a reduction in the expression of pro‐inflammatory genes, induction of genes involved in mitochondrial and energy metabolism, and alterations in the expression of genes involved in RNA metabolism. A metabolomic analysis has confirmed that gene expression changes across strains in metabolic genes in the liver are associated with expected biochemical changes in tissue and plasma (Collino *et al*., 2013). The current study is the first to examine gene expression of multiple mouse strains and tissues in a single, highly controlled experiment and with the same DNA microarrays. The use of transcriptional profiles to study the effects of CR and to identify CRMs does not require reliance on any specific pathway and has been previously used to show the overlap between the effects of CR and metformin in liver gene expression (Dhahbi *et al*., 2005) and the CR mimicry effects of resveratrol (Barger *et al*., 2008b), or a mixture of nutraceuticals containing resveratrol and quercetin (Barger *et al*., 2008a). More recently, this transcriptional approach was used to identify CRMs using a bioinformatics platform targeting modulation of CR‐induced transcriptional networks (Calvert *et al*., 2016). We opted to undergo an unbiased selection of robust transcriptional markers of CR, with the only selection criteria being consistency of effect on multiple mouse strains to avoid strain‐specific effects. Indeed, the identified genes represent a wide variety of biological processes, and most identified genes lack a clear functional link to CR at this time. It is noteworthy that there is minimal overlap between markers identified in different tissues (skeletal muscle, WAT, and heart), suggesting that the effects of CR (or at least its mediators) vary widely among different tissues. However, some general themes can be identified. It has been proposed that CR acts in part through depletion of intracellular acetyl‐CoA, which in turn induces autophagy (Mariño *et al*., 2014). Indeed, the highest level of induction of a CR transcriptional marker was observed for Acyl‐CoA synthetase short‐chain family member 2 (Acss2) in WAT (13‐fold induction), a gene involved in the production of intracellular acetate (Schug *et al*., 2015). Another transcriptional marker of CR in WAT is acetyl‐CoA carboxylase α (ACACA, 8.7‐fold induction with CR). In gastrocnemius muscle, we observed a reduction in the expression of acyl‐CoA‐thioesterase 2 (Acot2, 4.6‐fold), which localizes to the mitochondrial matrix and hydrolyzes long‐chain fatty acyl‐CoA into free fatty acids (FA) and CoASH, facilitating mitochondrial fatty acid oxidation (Moffat *et al*., 2014). Another transcriptional marker of CR in gastrocnemius muscle was malonyl‐CoA‐decarboxylase (Mlycd, −2.4‐fold change). Malonyl‐CoA‐decarboxylase regulates fatty acid biosynthesis and converts malonyl‐CoA to acetyl‐CoA. In heart, we observed reduced expression of stearoyl‐coenzyme A desaturase 4 (Scd4). SCD4 is a central enzyme in lipid metabolism that synthesizes monounsaturated fatty acids, and recent studies show that SCD4 has the ability to reprogram cardiac metabolism, thereby regulating heart function (Dobrzyn *et al*., 2015). It appears likely that alterations in tissue‐specific or organelle‐specific levels of acetyl‐CoA play an important role in these observed alterations in gene expression and may indeed be a central mechanism linking nutrient depletion, changes in gene expression, and long‐term health benefits of CR as previously proposed (Mariño *et al*., 2014).

One limitation of this study is the fact that the different mouse strains studied are likely to respond differently to CR as far as longevity or healthspan benefits. The DBA/2J strain in particular has been shown to have a shortened lifespan under some CR protocols (Fernandes *et al*., 1976; Forster *et al*., 2003), but not in others (Turturro *et al*., 1999). Thus, although our study has identified common transcriptional changes induced by CR in multiple mouse strains, the study does not address the long‐term effects of these transcriptional benefits on health and longevity outcomes in individual strains. Presumably, genetic background will have strong effects on whether specific transcriptional patterns lead to long‐term health benefits or not. Another limitation of our study is that we did not observe large‐scale CR‐induced changes in transcription in the brain neocortex, with only 22 genes changed in common in 3/7 strains. Furthermore, the fold change in expression of these 22 genes was inconsistent across strains (increased in some strains and decreased in other strains). These observations suggest that the brain, at least in young animals, is resistant to CR at the transcriptional level.

Following identification of these transcriptional markers of CR, we sought to use them to evaluate different phytochemical and pharmacological interventions thought to act as CRMs. Agents and doses were selected from the literature, and represent different categories of CRMs including modulators of mitochondrial metabolism (2,4‐dinitrophenol), glucose metabolism (pioglitazone, metformin), lipid metabolism (L‐carnitine, bezafibrate), and plant‐derived phytochemicals previously shown to mimic some of the health benefits of CR through multiple mechanisms (resveratrol, quercetin). Surprisingly, resveratrol, a compound previously reported to act as a CRM, did not appear to be strongly active, and in fact was not as active as the bioflavonoid quercetin. This may be due to the fact that in the current study a high dose of synthetic resveratrol was used. Alternatively, it may be that resveratrol has no effect in young mice, whereas it has been shown to exhibit CR mimicry in middle‐aged mice when fed at a much lower dose, from 14 to 30 months of age (Barger *et al*., 2008b). We previously reported that a nutraceutical mixture containing an even lower dose of resveratrol plus inositol hexaphosphate (IP6) and quercetin mimics gene expression profiles of CR in young mice fed from 2 to 5 months of age using a similar model (Barger *et al*., 2008b). In contrast, pioglitazone, an agonist of the lipid‐activated nuclear receptor PPARγ, and L‐carnitine, an amino acid involved in lipid metabolism, showed the strongest CR mimetic activity. We note that a previous study reported that agonists of lipid‐activated nuclear receptors are mimetics of CR (Corton *et al*., 2004). In that study, gene expression profiling of liver showed significant overlap between CR and agonists of lipid‐activated nuclear receptors. The beneficial effects of CR on liver protection from thioacetamide toxicity were shown to be dependent on lipid‐activated nuclear receptors, as shown with studies with PPAR‐α null mice (Corton *et al*., 2004). Our results extend these findings to additional tissues, indicating that lipid‐activated nuclear receptors may provide broad mimicry of CR. Indeed, pioglitazone not only impacted a large number of transcriptional markers of CR in a manner consistent with CR mimicry but also decreased adipocyte size, increased mitochondrial biogenesis as determined by citrate synthase activity, and reduced the level of the blood inflammatory marker IFN‐γ We note that pioglitazone increased blood levels of the inflammatory marker TNFα, which may be the result of toxicity. Surprisingly, the CR mimicry effects were observed despite no effect on glycated hemoglobin, a marker of blood glucose levels (Fig. 5A). Thus, one conclusion from this study is that many changes in gene expression mediated by CR do not require alterations in glucose levels.

L‐carnitine, an amino acid involved in lipid metabolism, altered the expression of 14/28 transcriptional markers of CR in a manner consistent with CR mimicry, and no markers were altered in a direction opposite of CR. Furthermore, L‐carnitine decreased adipocyte size and did not negatively impact inflammatory markers. L‐carnitine supplementation has previously been shown to improve markers of mitochondrial and metabolic function in aged rodents (Hagen *et al*., 1998; Noland *et al*., 2009) and has also been shown to improve some biochemical and functional parameters in the elderly (Malaguarnera *et al*., 2007; Badrasawi *et al*., 2016). In the heart, L‐carnitine appeared to have stronger activity than pioglitazone, as it altered the expression of three transcriptional markers of CR in a manner consistent with CR mimicry. Pioglitazone did not alter the expression of any transcriptional marker of CR in the heart in a manner consistent with CR mimicry. Thus, the activity of CRMs may be tissue specific, suggesting that combinations of CRMs may provide the best approach to achieve CR mimicry in multiple tissues.

We have previously shown that SIRT3, the major mitochondrial deacetylase, mediates at least some of the health benefits of CR in retarding aging (Someya *et al*., 2010) and that it does so by modulating energy metabolism, such as the urea cycle and fatty acid metabolism (Hallows *et al*., 2011), and enhancing the activity of enzymes that are involved in the antioxidant network, such as mitochondrial IDH2 (Someya *et al*., 2010). These effects may be a direct consequence of a mitochondrial energy metabolism reprogramming associated with the deacetylation of key mitochondrial proteins in response to higher Sirt3 levels induced upon CR. Surprisingly, none of the tested CRMs appeared to act by Sirt3 induction, as levels of Sirt3 in liver were not increased by these interventions. In fact, it is surprising that the levels of Sirt3 are actually reduced in liver by some of these compounds (Fig. 4B,C). It remains to be determined what molecular signal leads to the induction of Sirt3 upon CR. As CR was the only intervention that lowered glucose levels and is the only intervention that altered the organismal energetic balance through reduced food intake, it is possible that Sirt3 levels are a true reflection of the bioenergetic state of the cell and will not be easily modulated by CRMs (in the absence of CR). Interestingly, CS activity, a marker of mitochondrial mass and biogenesis, was induced by both CR and pioglitazone in adipose tissue (Fig. 4A). Therefore, increased mitochondrial mass induced by a CRM appears to be uncoupled from Sirt3 activation.

## Conclusions

There is growing interest in the identification of CRMs as a strategy to delay aging and increase human healthspan. Deciding what pathways or physiological assays are best suited for screening CRMs *in vivo* is challenging, as CR induces a large number of physiological adaptations, which may be tissue specific. We have identified panels of transcriptional markers of CR that are altered in expression by CR in multiple mouse strains in three important tissues (skeletal muscle, heart, and WAT). Importantly, we have shown that a drug that has strong activity in modulating CR transcriptional markers (pioglitazone) also modulates physiological measures of CR, such as reduced adipocyte size and mitochondrial mass. However, pioglitazone increased the levels of the inflammatory marker TNF‐α, a finding suggestive of drug side effects. The putative CR mimetic L‐carnitine, an amino acid involved in lipid metabolism, exhibited even stronger effects on CR transcriptional markers while modulating adipocyte size in a manner consistent with CR mimicry. These findings support the use of tissue‐specific, robust transcriptional markers of CR as an effective approach to screen and identify compounds that have the potential to mimic the beneficial effects of CR on lifespan and healthspan. We also note that based on the finding that different compounds display tissue‐specific CRM activity, it appears likely that stronger CR mimicry at the organismal level may be achieved by combining different CRMs.

## Experimental procedures

### Mice and diets

Male mice were purchased from Jackson Laboratories (Bar Harbor, USA) at 6 weeks of age and were individually housed in a pathogen free facility. From 6 to 8 weeks of age, mice were fed an AIN93M diet as one‐gram dustless precision pellets at a rate of 7/7/10 grams of food on Monday/Wednesday/Friday. At 8 weeks of age, mice were either maintained on the control diet or were assigned to a CR diet (see Supplemental methods for feeding details). For the studies of putative CR mimetics, male C57BL6/J mice were fed a control, CR, or a control diet supplemented with one of seven compounds from 8 to 22 weeks of age: 2,4‐dinitrophenol (24DNP; 1021 mg kg^−1^ diet; Sigma‐Aldrich, St. Louis, MO, USA); bezafibrate (BEZA; 100 mg kg^−1^ diet; Sigma‐Aldrich); metformin (METF; 1909 mg kg^−1^ diet; Sigma‐Aldrich); pioglitazone (PIOG; 100 mg kg^−1^ diet; Toronto Research Chemicals, Toronto, ON, Canada); L‐carnitine (1799 mg kg^−1^ diet; Lonza, Basel, Switzerland); quercetin (QUER; 510 mg kg^−1^ diet; Sigma‐Aldrich) Q4591); or trans‐resveratrol (RESV; 510 mg kg^−1^ diet; Sigma‐Aldrich). Doses of compounds to be included in diets were generally based on previous studies reported in the literature. 24DNP was based on the study of Reitman's group, which provided 800 mg L^−1^ of drinking water (Goldgof *et al*., 2014). Pioglitazone at 100 mg kg^−1^ diet resulted in a dosage of 10 mg kg^−1^ of body weight, a dose in between levels reported in two previous studies (Rodriguez *et al*., 2006; Thorp *et al*., 2007). Bezafibrate was initially fed at 500 mg kg^−1^ diet based on a previous study (Peters *et al*., 2003), but this dosage led to reduced food intake, and was subsequently reduced to 100 mg kg^−1^ diet. Metformin dosage was based on Dhahbi *et al*. (2005). Resveratrol dosage was based on Baur *et al*. (2006), and an identical dose of quercetin was used so that the activity of these two compounds could be compared. L‐carnitine dosage was partially based on a human study (Malaguarnera *et al*., 2007), and the equivalent mouse dose to a 1 g day^−1^ human daily intake was calculated using the method described in (Reagan‐Shaw *et al*., 2008).

At 22 weeks of age, mice were fasted overnight and euthanized by cervical dislocation and decapitation with collection of body cavity blood for determination of glycated hemoglobin. Tissues were collected and flash‐frozen in liquid nitrogen. Plasma inflammatory markers were measured in a separate set of mice that were treated identically, with the exception that mice were anesthetized by isoflurane inhalation and blood was collected by cardiac puncture. All procedures were approved by the Institutional Animal Care and Use Committee at the William S. Middleton Memorial Veteran's Hospital (Madison, USA).

### DNA microarray analysis and data filtering

For each of the seven strains, tissues from eight control and eight CR male mice were subjected to microarray analysis to identify transcriptional markers of CR in multiple strains**,** as described fully below and in the Data S1. RNA extraction, microarray analysis, and data preprocessing were performed as described previously (Barger *et al*., 2008b). Briefly, microarray analysis was performed using the Affymetrix Gene 1.0 ST array, and signal intensity data were generated from the Affymetrix Expression Console software using the gene‐level RMA‐Sketch algorithm (Irizarry *et al*., 2003). The resulting probe set data were filtered to remove probe sets not corresponding to a known gene, as well as probe sets corresponding to ambiguous and/or multiple genes. For those genes that were represented by multiple probe sets, we selected the probe set that had the largest signal intensity when averaged across the 16 arrays in that strain (eight control sample and eight CR samples for all strains and tissues, with the exception of DBA strain for which seven CR mice were used for heart analysis). The resulting data set yielded 20,067 unique transcripts that were used for analysis. The raw microarray data are freely accessible at: https://www.ncbi.nlm.nih.gov/geo/query/acc.cgi?token=claruggchnudtcj&acc=GSE75574

### Microarray data statistical analysis

A full description of statistical methods and analysis is reported in the Data S1. Briefly, custom scripts and Bioconductor tools (Huber *et al*., 2015) were written and deployed in RStudio (RStudio Team, 2015). Primary, strain‐specific gene lists were constructed by gene‐specific two‐sided *t*‐test *P*‐values < 0.01. The distribution on the number of genes predicted to exhibit a *t*‐test *P*‐value < 0.01 in multiple strains, assuming CR effects among strains are independent (null hypothesis), was computed as the sum of independent but not identically distributed Bernoulli trials, and thus subject to Poisson–Binomial sampling (Thomas & Taub, 1982; Data S1: Fig. SS‐2, Supporting information). False‐discovery‐rate control was assessed through a two‐way analysis of variance, combining CR and control data over all strains, within each tissue, and computing an FDR‐corrected *P*‐value for a main CR effect using Storey's *q*‐value (Storey, 2003; Data S1: Fig. SS‐3, Supporting information). Gene‐set enrichment analysis used 7108 GO terms annotating between 10 and 1000 mouse genes (Bioconductor annotation package org.Mm.e.g.db version 3.1.2) and random‐set enrichment computations as deployed in the R package *allez,* version 2.0.4 (Newton *et al*., 2007). Significance was declared at Bonferroni‐corrected *P*‐value 0.05 and required at least five CR‐associated genes within each identified GO term. Waterfall plots were constructed to visualize dominant GO terms (Figs 1 and S2–S4, Supporting information).

### Quantitative PCR

Quantitative RT–PCR was performed to confirm gene expression changes in a subset of genes, as well as to screen for the ability of interventions to mimic the effect of CR. We used the δ–δ Ct method to identify differential expression (Barger *et al*., 2008b), a complete list of qPCR primer assays for each gene is shown in Table S7 (Supporting information). We applied several criteria to select the subset of genes to be used in the qPCR analysis and subsequent screen of putative CR mimetics. When selecting genes, we gave preference to genes changed in a larger number of strains (e.g., a gene changed in 6/7 strains would be preferred over a gene changed in 5/7 strains), preference for a large fold change in expression in the majority of strains (<−1.4 or >1.4 on the microarray), and commercial availability of a quantitative RT–PCR (qPCR) primer assay. Although we did not specifically select genes on the basis of their known biological function, if there were multiple candidate genes with similar function, we selected the gene that more closely reflected the above selection criteria (e.g., *Tuba8* and *Tuba4a* were candidate genes in heart; however, we selected *Tuba8* for qPCR confirmation because it was changed in 6/7 strains and *Tuba4a* was changed in 5/7 strains). Genes meeting the above criteria were then analyzed for differential expression by qPCR in CO vs. CR C57BL6/J mice. For the CRM qPCR screen, C57BL6/J mice were used.

### SIRT3 abundance

SIRT3 abundance (*n *=* *8 mice per group) was measured in liver tissue by Western blotting using a SIRT3 rabbit monoclonal antibody (Cell Signaling Technology, Danvers, MA, USA). SIRT3 staining was measured by densitometry, and signal intensity was normalized to total protein staining on the blot.

### Citrate synthase activity

Citrate synthase activity (*n *=* *8 mice per group) was measured in WAT homogenates using the colorimetric method of Spinazzi *et al*. (2012).

### Adipocyte size

Adipocyte size (*n *=* *5–6 mice per group) was measured in formalin‐fixed WAT after hematoxylin and eosin staining. Sections were obtained from 5 to 6 mice per group, and adipocyte size was estimated by measuring 20–100 adipocytes per section.

### Glycated hemoglobin

Glycated hemoglobin (HbA1c (*n *=* *10 mice per group)) was measured using borate affinity ion‐exchange high‐performance liquid chromatography (Nuttall, 1998) at the Diabetes Diagnostic Laboratory at the University of Missouri Columbia (Columbia, SC, USA).

### Plasma cytokines

TNF‐α and IFN‐γ were measured in mouse plasma (*n *=* *10 mice per group) using the VPLEX Proinflammatory Panel from Meso Scale Diagnostics (Rockville, MD, USA).

### Other statistics

For qPCR assays, *t*‐tests were used to determine if a gene was differentially expressed in experimental vs. control‐fed mice (*P *<* *0.05). For all other measurements (body weight, adipocyte size, citrate synthase activity, SIRT3 abundance, HbA1c, and plasma cytokines), one‐way ANOVA was performed to evaluate if there was an overall effect of treatment; Dunnett's multiple comparison test was used for post hoc comparisons against control mice (*P *<* *0.05).

## Author contributions

JMV, NLC, and TDP performed experiments. AM, JLB, SMW, MAN, and SNH performed data analysis and interpretation. TAP, JLB, and RHW directed the research and wrote the manuscript.

## Conflict of interest

TAP and RW are cofounders of LifeGen Technologies, a company focused on developing interventions targeting the aging process. AM, SNH, and SMW are employed by Nu Skin Enterprises. TAP is a consultant to Nu Skin Enterprises and a member of its SAB. TAP also owns stock and is a consultant to CyteGen, a company focused on mitochondrial diseases.

## Funding

Research reported in this publication was partially supported by NIH grants R43AG034833 and RO1AG038679 to J.L.B and T.A.P, U54AI117924 to MAN, and by Nu Skin Enterprises.

## Supporting information


**Fig. S1** Average body weight at 22 weeks of age.
**Fig. S2** Graphical summary of gene set enrichment analysis of genes altered in expression in heart in response to CR.
**Fig. S3** Graphical summary of gene set enrichment analysis of genes altered in expression in gastrocnemius muscle in response to CR.
**Fig. S4** Graphical summary of gene set enrichment analysis of genes altered in expression in cerebral cortex in response to CR.

**Table S1** List of genes changed (*P *< 0.01) by CR in at least 4/7 mouse strains in heart tissue.

**Table S2** List of genes changed (*P *< 0.01) by CR in at least 5/7 mouse strains in gastrocnemius muscle.

**Table S3** List of genes changed (*P *< 0.01) by CR in at least 6/7 mouse strains in epididymal WAT.

**Table S4** List of genes changed (*P *< 0.01) by CR in at least 3/7 mouse strains in brain neocortex.

**Table S5** Inter‐strain and inter‐tissue variability in the response to CR at the gene expression level.

**Table S6** Reproducibility of qPCR.

**Table S7** TaqMan primer assay identifiers that were used for qPCR analysis.

**Data S1** Statistical supplement.
