# Supplementary material for: Identification of tissue‐specific transcriptional markers of caloric restriction in the mouse and their use to evaluate caloric restriction mimetics
Source: Aging Cell. 2017 May 26;16(4):750–60. doi: 10.1111/acel.12608 (PMC5506434; doi:10.1111/acel.12608)
Supplement: Supplementary file 1 — Fig. S1 Average body weight at 22 weeks of age. Fig. S2 Graphical summary of gene set enrichment analysis of genes altered in expression in heart in response to CR. Fig. S3 Graphical summary of gene set enrichment analysis of genes altered in expression in gastrocnemius muscle in response to CR. Fig. S4 Graphical summary of gene set enrichment analysis of genes altered in expression in cerebral cortex in response to CR. [file ACEL-16-750-s001.docx]

**Supporting Information**

**Supplemental Methods**

***Animal care and diets***

Details on calorie‑controlled feeding protocols have been described in detail elsewhere (Pugh et al., 1999). In this study, we adjusted the quantity of food provided for some strains based on previously‑observed body weight changes in response to CR. For the C3H/HeJ, CBA/J, DBA/2J and B6C3F1/J strains, we provided 7/7/10 grams of food on Monday / Wednesday / Friday to the CO mice and 5/5/8 grams of food (M/W/F) to the CR mice for the entire study (8-22 weeks of age). For the 129S1/SvImJ and C57BL/6J strains, CR mice received the same food allotment from 8-14 weeks of age, but starting at their 14th week, food allotment was further reduced to 4/4/6 grams of food (M/W/F). BALB/cJ rapidly lost body weight when provided a CR diet of 5/5/8 grams of food (M/W/F); accordingly, we adjusted food allotment in this strain such that CO mice received 9/9/13 grams and CR mice received 7/7/10 grams of food M/W/F.

***qPCR***

Aliquots of RNA were diluted to 20 ng μL^-1^ and gene expression was quantified using the TaqMan Gene Expression Assays shown in Table S7. RNA samples (*n*=8 mice per group) were analyzed in duplicate using EXPRESS One-Step SuperScript qRT-PCR Kit (Thermo Fisher Scientific, Waltham, USA) with total reaction volumes of 5 μL. The master mix contained 2.5 μL of EXPRESS qPCR SuperMix Universal, 0.5 μL of EXPRESS SuperScript Mix for One-Step qPCR, 0.25 μL of the appropriate TaqMan Gene Expression Assay and 0.15 μL of Ambion Nuclease-Free Water. The quantitative RT-PCR reactions were run on an Eppendorf Mastercycler ep realplex^2^ (Eppendorf North America, Hauppauge, USA). Threshold cycle (Ct) values were determined using Eppendorf software. The Ct for each sample was a calculation of the arithmetic mean of the duplicate values and changes due to the test diets were calculated using the δ‑δCt (delta-delta Ct) method (Barger et al., 2008a). To normalize for variations across assay plates, equal amounts of RNA from each control mouse (n=8) were used to make a pooled RNA sample, and four replicates of the pooled sample were analyzed on each plate. The average expression value of the pooled RNA from one plate was compared to the average expression of a second plate, and any difference in expression was used as a correction factor for all samples on the second plate.

***Western blotting***

Protein samples were extracted from approximately 20 mg of liver tissue using a Wheaton glass/glass tapered conical tissue grinder Powered by a SciLogex OS20-S overhead stirrer operated at 500 rpm (SciLogex, Berlin, USA). The extraction buffer, pH 7.9, consisted of 20 mM K-HEPES, 125 mM NaCl, 0.1% Igepal (NP-40), 0.1% Triton X-100, 1 mM EDTA, 1 μM Trichostatin A, 10 mM nicotinamide, and 1X protease/phosphatase inhibitors (Cell Signaling Technology Inc., Danvers, USA). Protein concentrations were determined using the Bio-Rad Quick-Start Bradford Assay Kit (Bio-Rad, Hercules, USA). Samples for electrophoresis consisted of 10 μg protein, 1X Laemlii sample buffer (Bio-Rad, Hercules, USA), 2.5% β-mercaptoethanol and water added to a final volume of 10 μl. Samples were heated to 70°C for 10 minutes and loaded on midi-size Bio-Rad Precast Criterion TGX Stain-free 4‑15% gels (Bio-Rad, Hercules, USA). Following electrophoresis, the gels were exposed to UV light for 45 seconds resulting in a covalent reaction between the trihalogen compound included in the gel and tryptophan residues in the protein samples. The resulting modified proteins fluoresced with exposure to UV light and were photographed using a BioSpectrum AC Imaging System (UVP, Upland, USA). Following transfer onto Trans-Blot Turbo Midi PVDF Transfer Packs (Bio-Rad, Hercules, USA), the intensity of protein staining in each lane of the blot was measured by densitometry using VisionWorks LS software (UVP) and served to correct for protein loading. Blots were washed for 5 min in TBST (20 mM Tris, 150 mM NaCl, 0.1% Tween 20, pH 7.6) then blocked in 5% BSA (Cell Signaling Technology Inc., Danvers, USA) in TBST with agitation for one hour. Blots were incubated overnight with agitation in SIRT3 Rabbit monoclonal anti-body (Cell Signaling Technology Inc., Danvers, USA) diluted 1/3,000 in 5% BSA in TBST. Blots were then washed 5 times for 5 minutes each in TBST and incubated with agitation for one hour in anti-rabbit poly-HRP secondary antibody (Thermo Fisher Scientific, Waltham, USA) diluted 1/15,000. Following the incubation in the secondary antibody, blots were washed six times for five minutes each in TBST, then incubated with agitation for five minutes in SuperSignal West Pico enhanced chemiluminescent substrate (Thermo Fisher Scientific, Waltham, USA) and finally exposed to CL-XPosure X-ray Film (Thermo Fisher Scientific, Waltham, USA). Films were scanned and intensity of SIRT3 staining was analyzed using Image J software ([http://rsb.info.nih.gov/ij](http://rsb.info.nih.gov/ij/)), NIH, Bethesda, MD, USA).

***Citrate synthase activity***

Approximately 50 mg of adipose tissue was homogenized in 20 volumes of homogenization buffer consisting of 250 mM sucrose, 20 mM Tris pH 7.4, 40 mM KCL and 2 mM EGTA. Homogenates were centrifuged at 600g for 10 min at 4°C. Supernatants were collected and protein concentration was determined using the Bio-Rad Quick-Start Bradford Assay Kit. Samples were then subjected to three rapid freeze-thaw cycles. We used the method of Spinazzi et al. (Spinazzi et al., 2012), which measures the reaction between acetyl coenzyme A (acetyl CoA) and oxaloacetic acid to form citric acid. The hydrolysis of the thioester of acetyl CoA generates a thiol group (CoA-SH) which reacts with 5,5′-Dithiobis-(2-nitrobenzoic acid) (DTNB) to form a product which is measured spectrophotometrically at 410 nm. Following subtraction of non-specific baseline activity, citrate synthase activity is calculated using the extinction coefficient for DTNB (13.6 mmol min^-1^ mg^-1^) and the data is expressed as mmols^-1^ min^-1^ mg protein^-1^. The reactions consisted of 100 mM tris pH 8.0, 0.1% Triton X-100, 10 μM DTNB, 30 μM acetyl CoA and sample homogenates containing 15 μg protein. Samples were measured in duplicate. Volumes were adjusted to 185.6 μl per well with water, then baseline activity was read at 410 nm in the plate reader at 37°C once per minute for 10 minutes. The plate was then chilled on ice for 5 minutes before addition of oxaloacetic acid to a final concentration of 0.72 mM and a final volume of 200 μl per well. The chilled plate was placed in the spectrophotometer at 37°C to warm for 5 minutes and absorbance at 410 nm was read once per minute for 10 minutes.

**Fig. S1. Average body weight at 22 weeks of age.** Body weight was significantly (*p*<0.01) lower in all strains in response to CR except for the BALB/cJ strain (*p*=0.42). Data represent means with standard error of the mean.

**
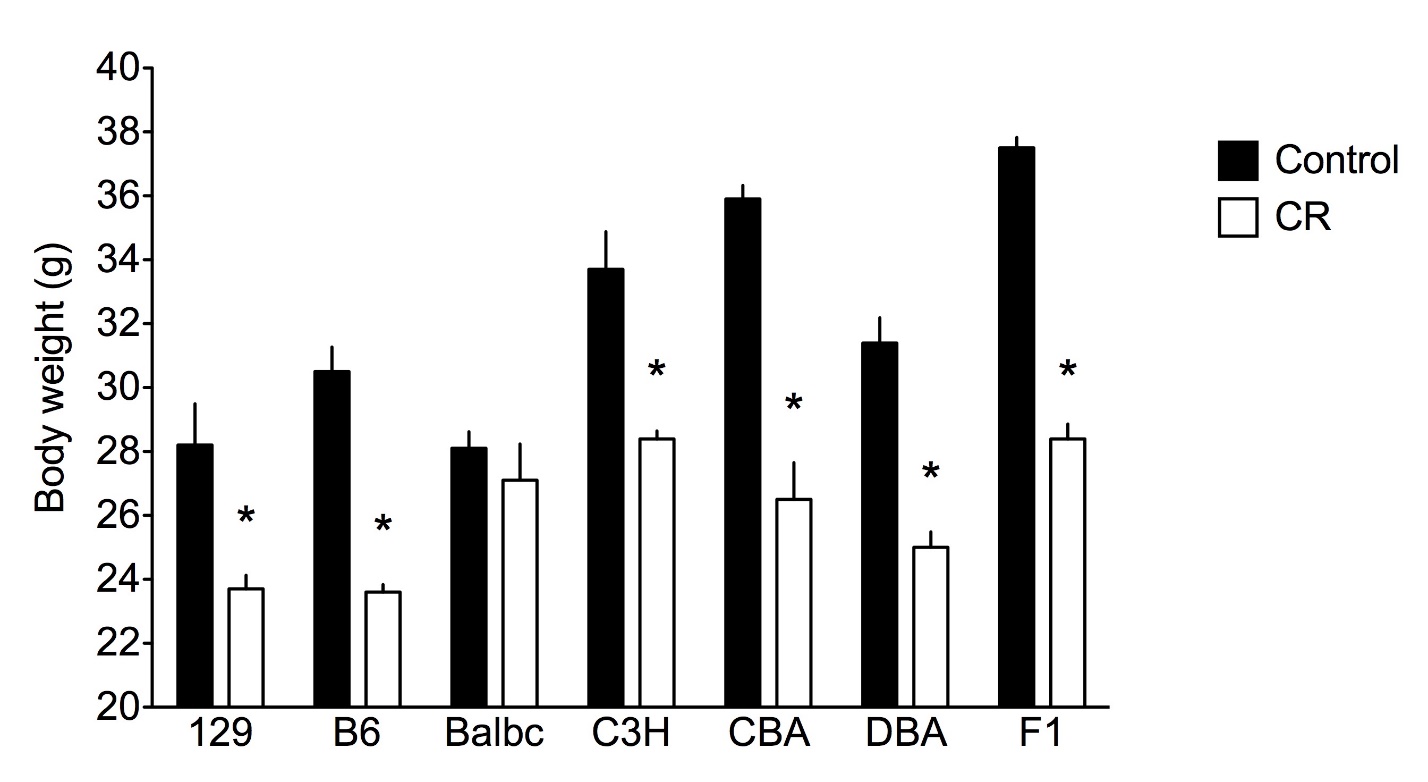
**

**Fig. S2. Graphical summary of gene set enrichment analysis of genes altered in expression in heart in response to CR**


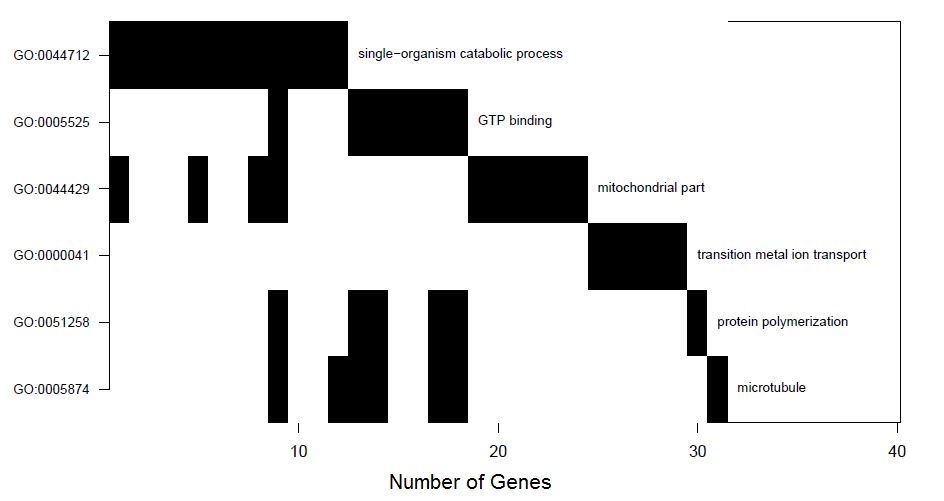


**Fig. S3. Graphical summary of gene set enrichment analysis of genes altered in expression in gastrocnemius muscle in response to CR**


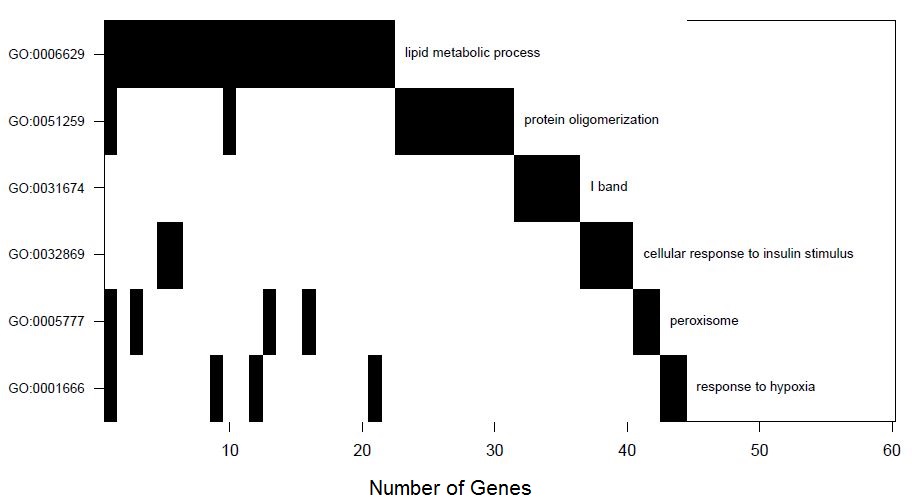


**Fig. S4. Graphical summary of gene set enrichment analysis of genes altered in expression in cerebral cortex in response to CR**


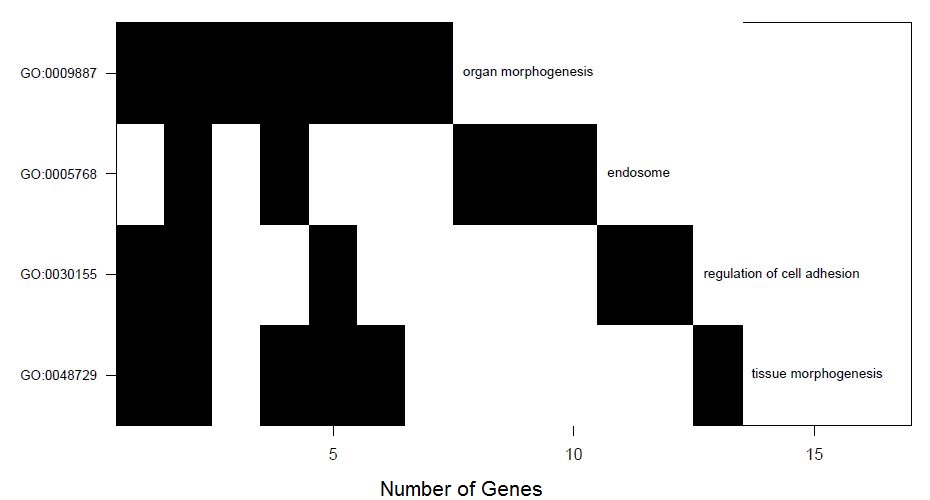


**Table S1.** List of genes changed (*p*<0.01) by CR in at least 4/7 mouse strains in heart tissue. Yellow or blue fill indicate genes that were significantly increased or decreased, respectively, in expression in response to CR. Genes indicated with “Y” in the “PCR” column represent those genes that were used for qPCR analysis.

**Table S2.** List of genes changed (*p*<0.01) by CR in at least 5/7 mouse strains in gastrocnemius muscle. Yellow or blue fill indicate genes that were significantly increased or decreased, respectively, in expression in response to CR. Genes indicated with “Y” in the “PCR” column represent those genes that were used for qPCR analysis.

**Table S3.** List of genes changed (*p*<0.01) by CR in at least 6/7 mouse strains in epididymal WAT. Yellow or blue fill indicate genes that were significantly increased or decreased, respectively, in expression in response to CR. Genes indicated with “Y” in the “PCR” column represent those genes that were used for qPCR analysis.

**Table S4.** List of genes changed (*p*<0.01) by CR in at least 3/7 mouse strains in brain neocortex. Yellow or blue fill indicate genes that were significantly increased or decreased, respectively, in expression in response to CR. Genes indicated with “Y” in the “PCR” column represent those genes that were used for qPCR analysis.

**Table S5.** Inter-strain and inter-tissue variability in the response to CR at the gene expression level. Each row represents the total number of genes changed in expression by CR (*p*<0.01).

**Table S6.** Reproducibility of qPCR. Each gene was tested in three independent cohorts of C57BL/6J mice subjected to the same CR extent and duration of CR (Biological replicates 1-3). Each gene was then assessed for technical variation by retesting two additional times with the same RNA sample (Biological replicates 3.2 and 3.3).

**Table S7.** TaqMan primer assay identifiers that were used for qPCR analysis.
