# Supplementary material for: Identification of tissue‐specific transcriptional markers of caloric restriction in the mouse and their use to evaluate caloric restriction mimetics
Source: Aging Cell. 2017 May 26;16(4):750–60. doi: 10.1111/acel.12608 (PMC5506434; doi:10.1111/acel.12608)
Supplement: Supplementary file 9 — Data S1 Statistical supplement. [file ACEL-16-750-s009.docx]

# Statistical Supplement ^[[1]](#footnote-1)^

## Effects of caloric restriction on gene expression

Version: 2017-02-15.

**Supplementary to primary manuscript:** Barger et al., *Identification of Tissue-specific Transcriptional Markers of Caloric Restriction in the Mouse and Their Use to Evaluate Caloric Restriction Mimetics.*

The gene-expression data analyses summarized in the primary manuscript are presented here in further detail. These include differential expression between CR treated and control mice in various tissues and mouse strains, and gene-set enrichment analysis.

**Data:**  Expression profiles (20067 transcripts, after pre-processing) were available for 8 CR and 8 control animals in each of 7 mouse strains, giving 112 profiles in each of four tissues (heart, gastrocnemius, adipose, and cortex), and thus, in total, 448 genome-wide expression profiles. Quality control checks identified one array in heart tissue as unusually outlying, and it was removed from subsequent computations.

**CR-effects within strain:** As a first test for CR effects we performed t-test calculations separately within each tissue type and within each mouse strain, computing gene specific, raw scale, two-sided, Student t-tests to compare CR with control expression. Shown below are the numbers of genes that have uncorrected t-test CR p-value less than 0.01, for all tissues and strains.

Table SS-1: Numbers of genes by t-test: CR vs control, p<0.01

|  | adipose | heart | gastrocnemius | cortex |
| --- | --- | --- | --- | --- |
| Balb | 235 | 333 | 929 | 353 |
| DBA | 1217 | 262 | 1301 | 345 |
| 129 | 5406 | 2126 | 1523 | 369 |
| C3H | 3174 | 426 | 1062 | 100 |
| F1 | 6058 | 1342 | 793 | 124 |
| B6 | 3634 | 813 | 1652 | 415 |
| CBA | 4148 | 348 | 1185 | 754 |
|  |  |  |  |  |

Table SS-2: As a percentage of total number of genes

|  | adipose | heart | gastrocnemius | cortex |
| --- | --- | --- | --- | --- |
| Balb | 1.2 | 1.7 | 4.6 | 1.8 |
| DBA | 6.1 | 1.3 | 6.5 | 1.7 |
| 129 | 26.9 | 10.6 | 7.6 | 1.8 |
| C3H | 15.8 | 2.1 | 5.3 | 0.5 |
| F1 | 30.2 | 6.7 | 4.0 | 0.6 |
| B6 | 18.1 | 4.1 | 8.2 | 2.1 |
| CBA | 20.7 | 1.7 | 5.9 | 3.8 |

Note that in the absence of any CR effects the expected percentage in each case above is only $1\%$. These calculations show the large extent of tissue and strain specific CR effects. They don't reveal how consistent are these effects across strains, which is considered next.

**Consistency across strains: the multi-t-test rule**

Tabulated here are the number of genes that have uncorrected t-test p-value less than 0.01 in various numbers of strains (rows).

Table SS-3: Significant CR vs control genes in multiple strains

|  | adipose | heart | gastrocnemius | cortex |
| --- | --- | --- | --- | --- |
| 0 | 9725 | 15901 | 14788 | 17854 |
| 1 | 4344 | 3077 | 3453 | 2000 |
| 2 | 2254 | 812 | 1038 | 185 |
| 3 | 1531 | 190 | 437 | 22 |
| 4 | 1056 | 62 | 208 | 6 |
| 5 | 744 | 19 | 90 | 0 |
| 6 | 408 | 6 | 48 | 0 |
| 7 | 5 | 0 | 5 | 0 |

We consider several selection rules to identify genes showing relatively consistent effects across strains. First, the *multi-t-test* rule asks that a gene have CR p-value less than 0.01 in at least $k/7$ strains, where we allow $k$ to vary owing to differences among tissues in global CR effects. We use $k=6$ (adipose), $k=4$ (heart) and $k=5$ (gastrocnemius), and thus identify 413 genes in adipose, 87 genes in heart, and 143 in gastrocnemius. The CR signal is much weaker in cortex; we would find 28 using $k=3$. Details on the genes with strong, consistent CR effects by the multi-t-test rule are shown in Tables S1-S4. We note that the genes selected by the multi-t-test rule are generally expressed above background levels (next section), and also that the gene lists carry a low false discovery rate (following sections).

**Expression relative to background:** Genes identified by the multi-t-test rule have expression levels that are relatively high compared to the rest of the transcriptome. The panels in Figure SS-1 show for each tissue the distribution (grey) of expression levels per gene (log2 scale, averaged over all arrays in that tissue). Highlighted (red) are histograms of the genes selected by the multi-t-test rule. Quantifying the relative expression levels in adipose, for example, 403, of the 413 selected genes exceed in average expression the median (over genes) average (over arrays) expression of non-selected genes.


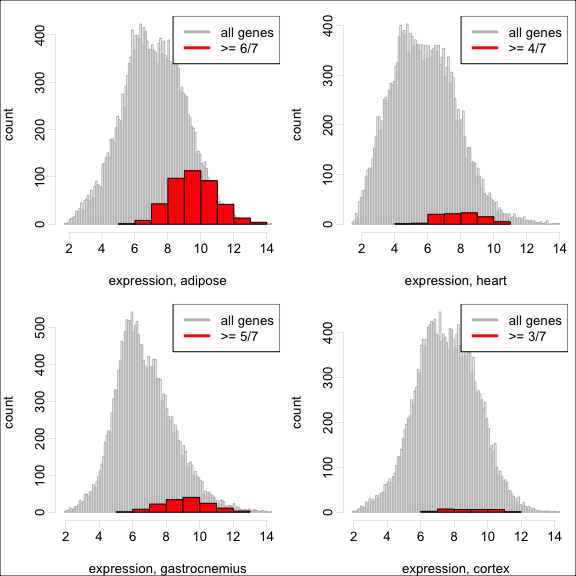


Figure SS-1: Average expression levels of all genes (grey) and genes selected by multi-t-test rule (red)

**Directional consistency:** CR effects are consistent in the sense that genes identified by the multi-t-test rule are usually either up by CR in all strains showing a significant change, or down by CR in all strains showing such change. Recall that the mult-t-test selection rule required genes to have an uncorrected two-sided t-test p-value less than $0.01$ in sufficiently many strains. We did not enforce directional consistency in the selection of genes, although the selected genes do exhibit this consistency, as the table below shows:

Table SS-4: Numbers of selected genes and numbers of genes showing complete consistency (either up in all strains where significant, or down in all strains where significant)

|  | completely consistent selected genes | number selected genes |
| --- | --- | --- |
| adipose | 412 | 413 |
| heart | 86 | 87 |
| gastrocnemius | 139 | 143 |
| cortex | 24 | 28 |

**Multiplicity correction:** Though the p-values driving the multi-t-test selection are not corrected for multiple comparisons, their consistency over strains delivers false discovery rate control. We show two calculations in support of this claim.

**Multiple-strain t-test agreement:** An important issue concerns how likely it is to have a gene with a p-value less than 0.01 in at least $k/7$ strains, under the hypothesis that CR effects are unrelated among strains. The second table on page 1 shows the percentage of genes with p-value less than 0.01 over various strains and tissues. We treat these as strain-specific rates defining the chances that a gene would have a strong p-value in each strain. In most tissues, for example, many more than $1\%$ of genes have p-value less than $1\%$ in a given strain; indeed one may compute the chances that a gene would show up on multiple $p\leq0.01$ lists. If CR effects are independent among strains (null hypothesis), then the number of strains for which a given gene would yield p-value less than 0.01 is the sum of independent but not-identical Bernoulli trials, and therefore has a Poisson-Binomial distribution (Thomas and Taub, 1982), shown in Figure SS-2. This calculation advances the binomial computation used for a similar purpose in De Magalhães et al., 2007.

If CR effects are independent among strains, the probability that a gene appears on multiple CR lists is quite small. For example in adipose, we expect 0.821 genes in the genome to meet the selection criteria, though 413 are in fact selected by the $6/7$ rule.


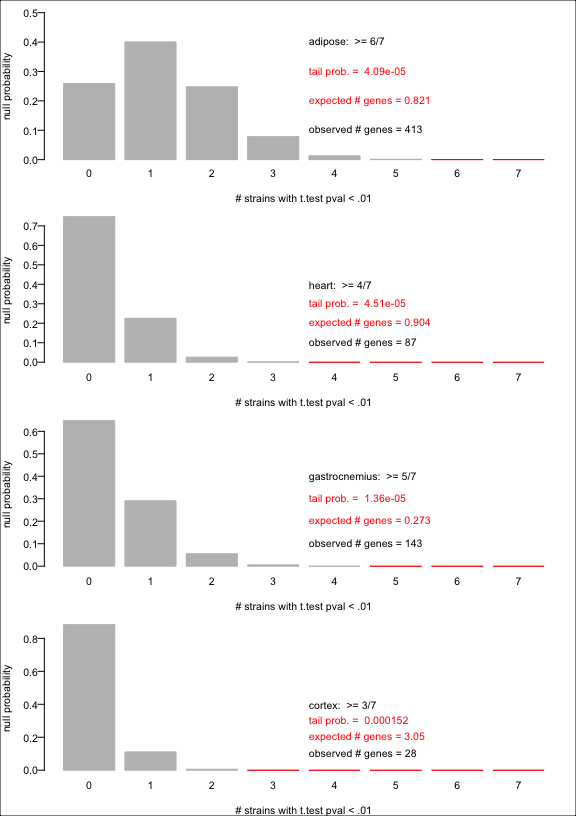


Figure SS-2: Null probability distribution of the number of strains for which a gene has CR p-value less than 0.01

**Consistent CR effects: ANOVA**. It is useful to control the false-discovery-rate (FDR) of any list of genes purported to have a certain effect, such as differential expression between CR and control animals. Though the mult-t-test rule does not lend itself to automatic FDR control, we can apply two-way analysis of variance (ANOVA) per gene to analyze profiles from all strains at once in a given tissue. This combined analysis produces gene-specific FDR estimates for *across-strain* CR effects. Briefly, we decompose the log2 expression level of each gene on each array as an overall mean plus a strain effect plus a CR effect plus an interaction term plus a noise term, and we obtain the best decomposition via least squares. All arrays are processed simultaneously (rather than strain by strain). The interaction terms measure how the CR vs control differences fluctuate over strains, while the main CR effect records an average *across strain* effect of caloric restriction. Figure SS-3 shows negative log10 p-values for CR effect as a function of the results t-test results from the earlier analysis. The across-strain, main CR-effect p-values can be corrected for false discovery rate control (we use the q-value calculator, Storey, 2003). Supporting the multi-t-test selection scheme, all selected genes in heart, adipose, and gastrocnemius have ANOVA-FDR-corrected p-value much less than 0.05.


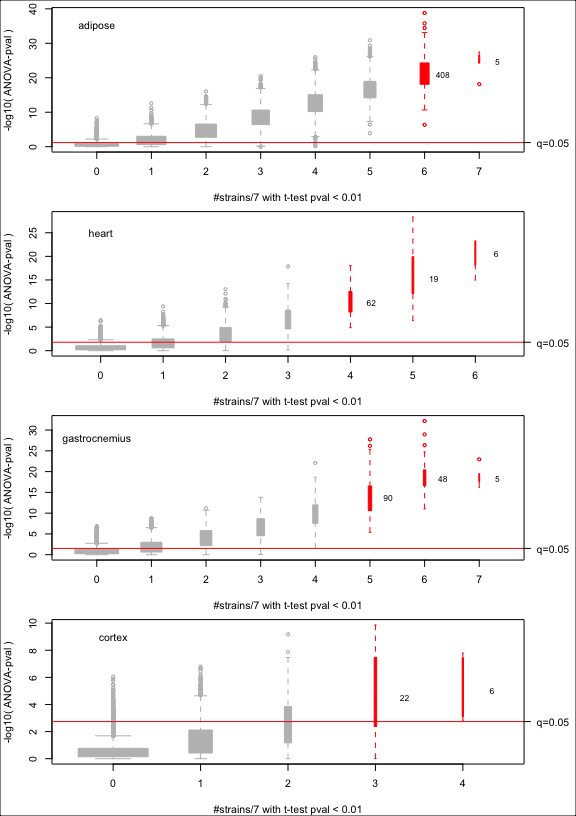


Figure SS-3: Analysis of variance CR p-value versus multi-t-test results; q=0.05 threshold corresponds to 5% FDR corrected p-value

**Across-strain CR effects** Figure SS-4 displays the selected genes through *volcano* plots, which relate the p-value for the across-strain CR effect (vertical) with the estimated fold effect (CR vs control), both from the ANOVA calculation. The multi-t-test selected genes identified in red.


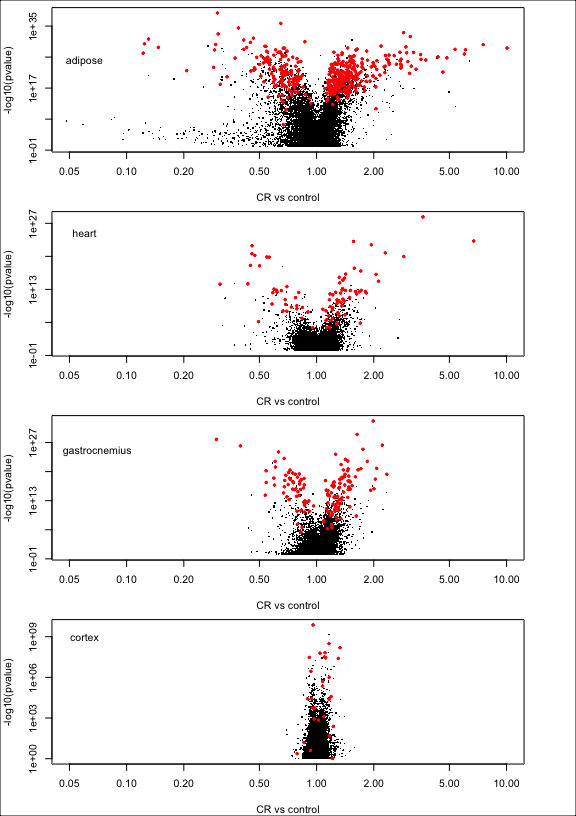


Figure SS-4: Volcano plots: CR significance versus fold effects from two-way ANOVA. Genes selected by multi-t-test rule are shown in red

Next we report a series of heatmaps (blue=low, red=high) that show expression data on all mice, after adjusting for strain differences, and for the genes selected by the mult-t-test rule (Figures SS-5 to SS-8).


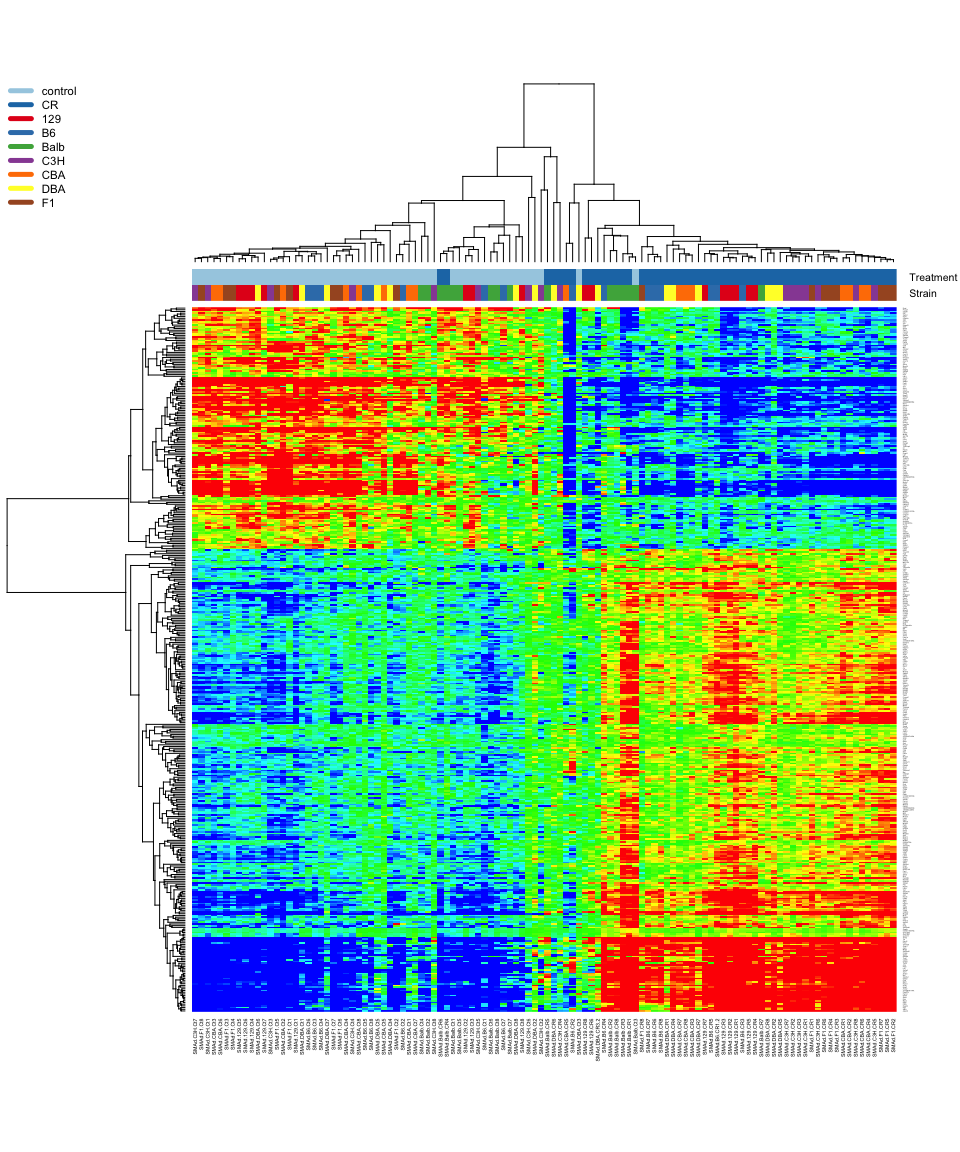


Figure SS-5: Strain-adjusted CR gene expression, adipose


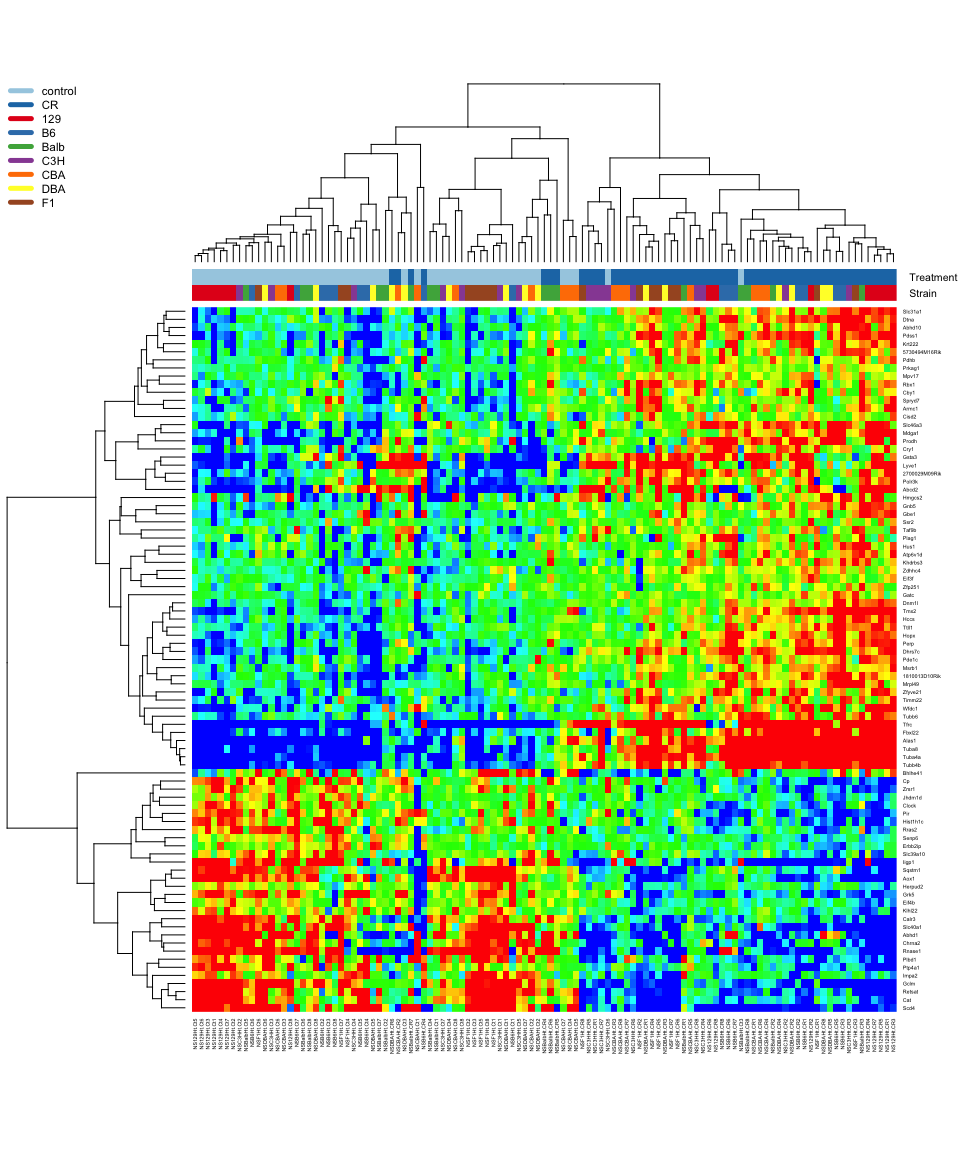


Figure SS-6: Strain adjusted CR gene expression, heart


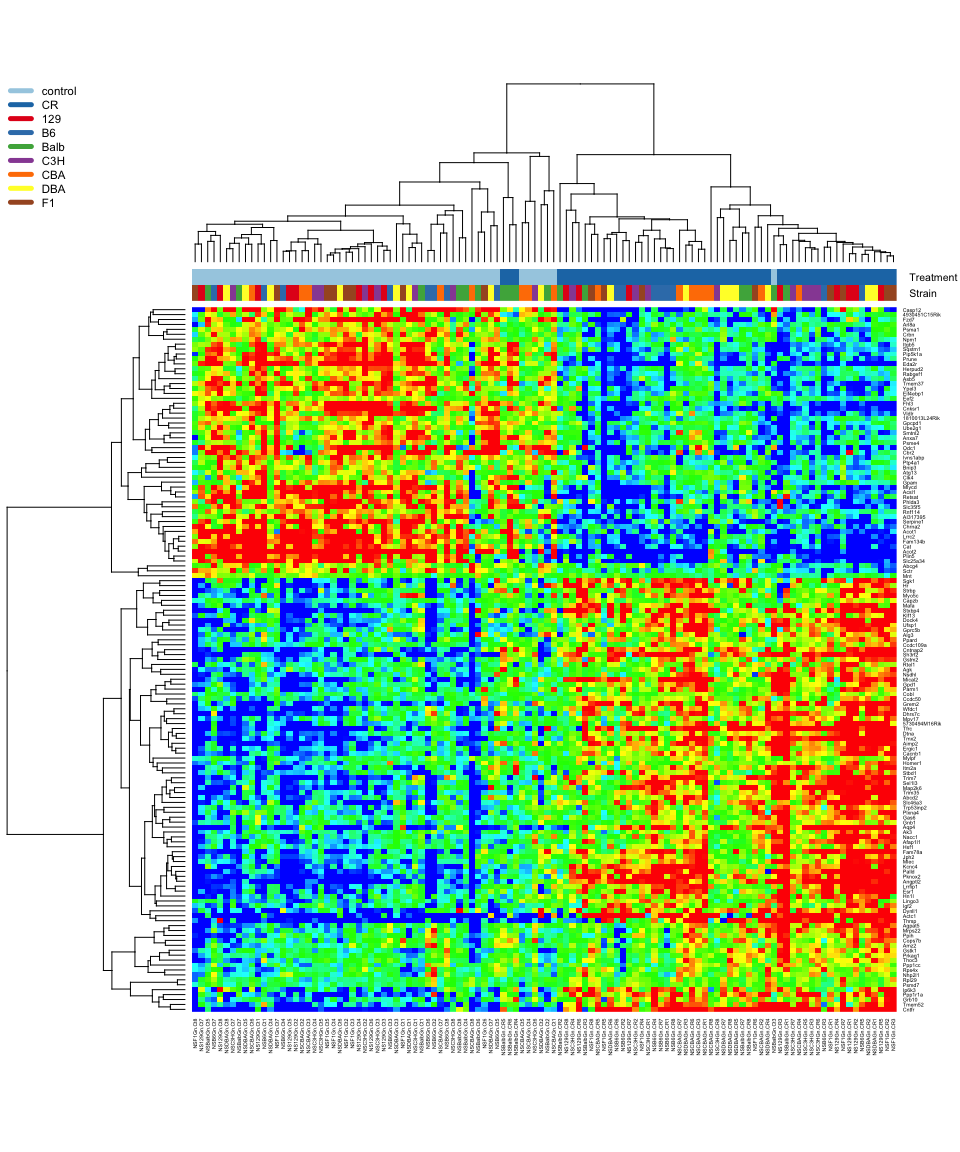


Figure SS-7: Strain adjusted CR gene expression, gastrocnemius


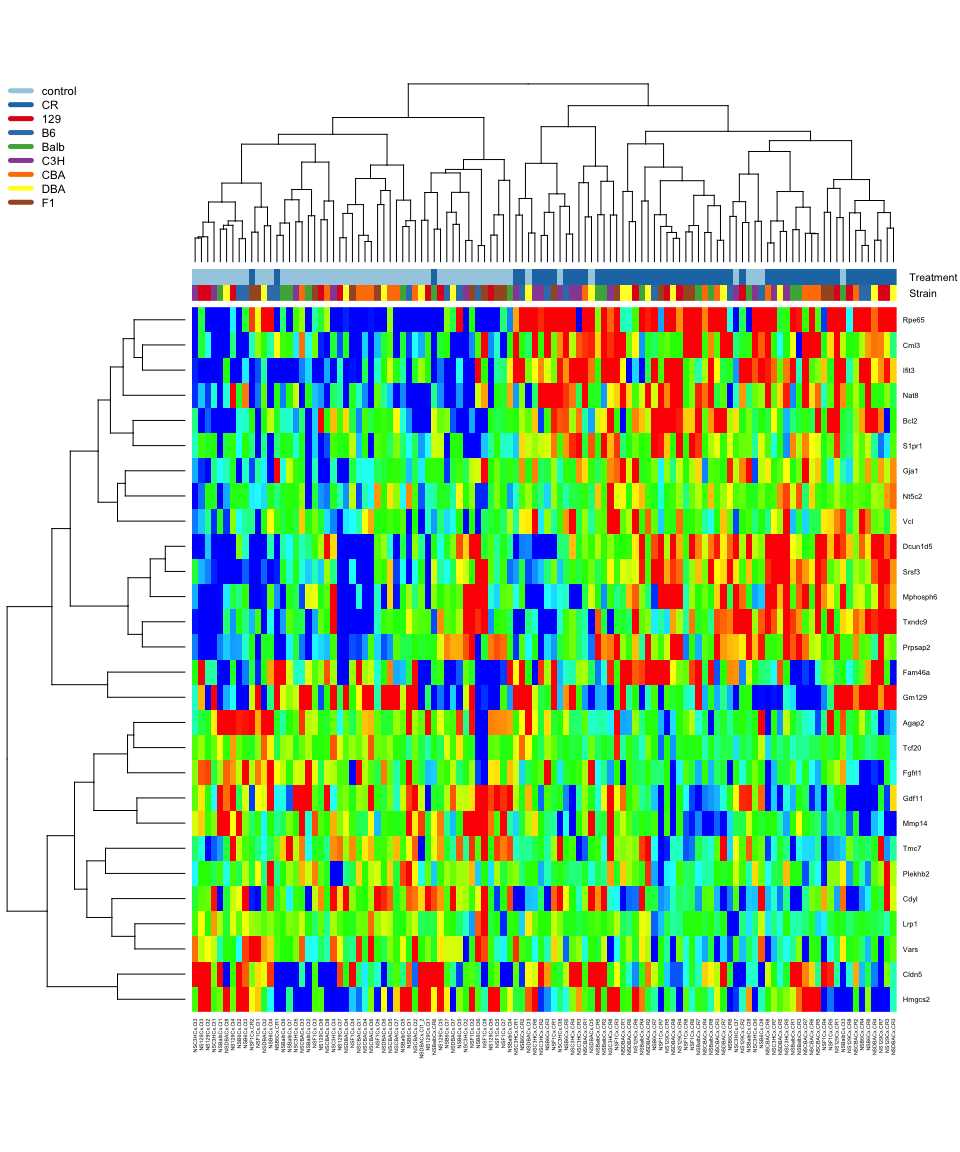


Figure SS-8: Strain adjusted CR gene expression, cortex

## Gene set enrichment

We investigated the functional content of gene lists identified by the mult-t-test rule using random-set enrichment methods encoded in the *allez* R package and applied to the Gene Ontology (Newton et al. 2007). For the four tissues, Tables SS-5 through SS-8 show the GO terms that are significantly enriched for CR-multi-t-test genes, at Bonferonni-corrected p-value of 0.05 (z score larger than 4.34), and based on the library of 7108 gene sets (GO terms) annotating between 10 and 1000 genes. Graphical summaries of this calculation are shown in the following figures. In these *waterfall* plots, we find the significant GO term having largest overlap with the CR gene list (e.g., in adipose, it is the cellular component <GO:0044429>, *mitochondrial part*, which explains 71 CR genes), and note that in the top row of the figure. Then we remove those genes from the CR list and find the significant GO term having highest overlap with the remainder (in adipose, this is <GO:0019637>). The process is repeated as long as new significant gene sets describe at as-yet-undescribed CR genes. Genes identified by this sequential process are counted along the x-axis. Owing to extensive overlap among GO terms, we note that more GO terms than shown in these plots are significantly enriched (i.e. the waterfall shows a subset of the 150 GO terms from Table SS-5); the sequential coverage calculation identifies a dominant set of significant GO terms for display. See, e.g. (Hao et al. 2015) for other examples of this plotting scheme.


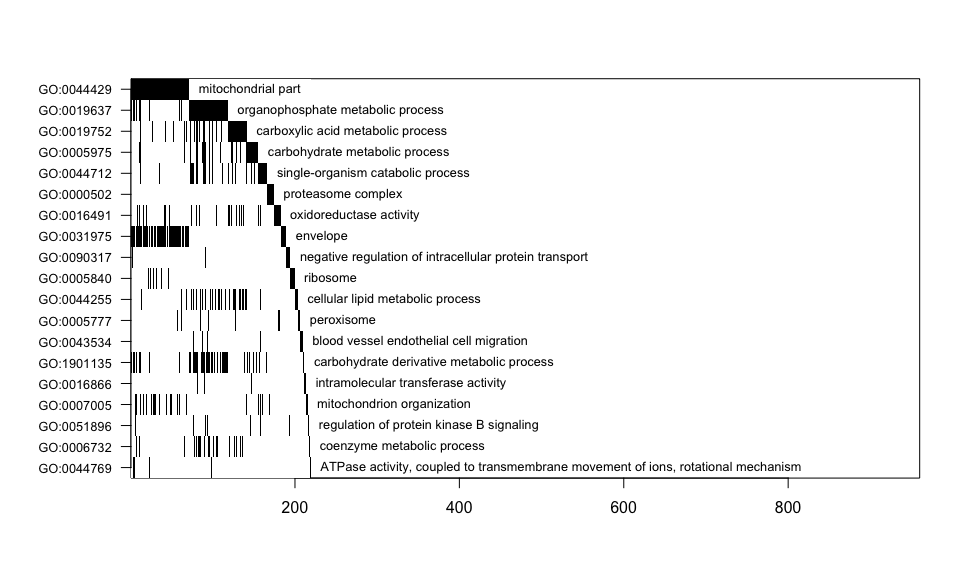


Table SS-5: Gene Ontology terms enriched for multi-t-test CR genes, adipose; set.mean is proportion of GO term that is CR related

|  | Term | Ontology | CRgenes | set.size | set.mean | z.score |
| --- | --- | --- | --- | --- | --- | --- |
| <GO:0044429> | mitochondrial part | CC | 71 | 592 | 0.12 | 16.65 |
| <GO:0005743> | mitochondrial inner membra... | CC | 48 | 331 | 0.15 | 15.51 |
| <GO:0005740> | mitochondrial envelope | CC | 57 | 481 | 0.12 | 14.74 |
| <GO:0019866> | organelle inner membrane | CC | 48 | 364 | 0.13 | 14.55 |
| <GO:0031966> | mitochondrial membrane | CC | 53 | 446 | 0.12 | 14.23 |
| <GO:0031975> | envelope | CC | 67 | 755 | 0.09 | 12.89 |
| <GO:0031967> | organelle envelope | CC | 66 | 752 | 0.09 | 12.67 |
| <GO:0006084> | acetyl-CoA metabolic proce... | BP | 9 | 25 | 0.36 | 11.60 |
| <GO:0009156> | ribonucleoside monophospha... | BP | 14 | 59 | 0.24 | 11.37 |
| <GO:0044455> | mitochondrial membrane par... | CC | 21 | 126 | 0.17 | 11.19 |
| <GO:0009127> | purine nucleoside monophos... | BP | 13 | 54 | 0.24 | 11.05 |
| <GO:0009168> | purine ribonucleoside mono... | BP | 13 | 54 | 0.24 | 11.05 |
| <GO:0019637> | organophosphate metabolic ... | BP | 58 | 744 | 0.08 | 10.73 |
| <GO:0009124> | nucleoside monophosphate b... | BP | 14 | 65 | 0.22 | 10.72 |
| <GO:0006090> | pyruvate metabolic process... | BP | 15 | 76 | 0.20 | 10.52 |
| <GO:0006091> | generation of precursor me... | BP | 28 | 235 | 0.12 | 10.30 |
| <GO:1990204> | oxidoreductase complex | CC | 14 | 72 | 0.19 | 10.07 |
| <GO:0006732> | coenzyme metabolic process... | BP | 24 | 191 | 0.13 | 9.90 |
| <GO:0009161> | ribonucleoside monophospha... | BP | 17 | 106 | 0.16 | 9.81 |
| <GO:0009145> | purine nucleoside triphosp... | BP | 10 | 42 | 0.24 | 9.62 |
| <GO:0006753> | nucleoside phosphate metab... | BP | 39 | 448 | 0.09 | 9.59 |
| <GO:0005747> | mitochondrial respiratory ... | CC | 9 | 35 | 0.26 | 9.56 |
| <GO:0030964> | NADH dehydrogenase complex... | CC | 9 | 35 | 0.26 | 9.56 |
| <GO:0045271> | respiratory chain complex ... | CC | 9 | 35 | 0.26 | 9.56 |
| <GO:0009167> | purine ribonucleoside mono... | BP | 16 | 101 | 0.16 | 9.44 |
| <GO:0009117> | nucleotide metabolic proce... | BP | 38 | 440 | 0.09 | 9.40 |
| <GO:0051186> | cofactor metabolic process... | BP | 26 | 235 | 0.11 | 9.40 |
| <GO:0009126> | purine nucleoside monophos... | BP | 16 | 102 | 0.16 | 9.38 |
| <GO:1901135> | carbohydrate derivative me... | BP | 54 | 778 | 0.07 | 9.31 |
| <GO:0009123> | nucleoside monophosphate m... | BP | 17 | 115 | 0.15 | 9.30 |
| <GO:0055086> | nucleobase-containing smal... | BP | 40 | 487 | 0.08 | 9.26 |
| <GO:0009144> | purine nucleoside triphosp... | BP | 15 | 93 | 0.16 | 9.25 |
| <GO:0009163> | nucleoside biosynthetic pr... | BP | 14 | 84 | 0.17 | 9.13 |
| <GO:0042455> | ribonucleoside biosyntheti... | BP | 14 | 84 | 0.17 | 9.13 |
| <GO:1901659> | glycosyl compound biosynth... | BP | 14 | 84 | 0.17 | 9.13 |
| <GO:0042451> | purine nucleoside biosynth... | BP | 13 | 74 | 0.18 | 9.10 |
| <GO:0046129> | purine ribonucleoside bios... | BP | 13 | 74 | 0.18 | 9.10 |
| <GO:0019693> | ribose phosphate metabolic... | BP | 30 | 325 | 0.09 | 8.80 |
| <GO:0005746> | mitochondrial respiratory ... | CC | 10 | 49 | 0.20 | 8.76 |
| <GO:0006754> | ATP biosynthetic process | BP | 8 | 33 | 0.24 | 8.70 |
| <GO:0009206> | purine ribonucleoside trip... | BP | 9 | 41 | 0.22 | 8.69 |
| <GO:0070469> | respiratory chain | CC | 11 | 60 | 0.18 | 8.59 |
| <GO:0005759> | mitochondrial matrix | CC | 19 | 160 | 0.12 | 8.45 |
| <GO:0009201> | ribonucleoside triphosphat... | BP | 9 | 43 | 0.21 | 8.44 |
| <GO:0009116> | nucleoside metabolic proce... | BP | 20 | 175 | 0.11 | 8.43 |
| <GO:0009141> | nucleoside triphosphate me... | BP | 15 | 108 | 0.14 | 8.37 |
| <GO:0009142> | nucleoside triphosphate bi... | BP | 10 | 53 | 0.19 | 8.34 |
| <GO:0043209> | myelin sheath | CC | 20 | 178 | 0.11 | 8.33 |
| <GO:0015985> | energy coupled proton tran... | BP | 5 | 15 | 0.33 | 8.28 |
| <GO:0015986> | ATP synthesis coupled prot... | BP | 5 | 15 | 0.33 | 8.28 |
| <GO:0009205> | purine ribonucleoside trip... | BP | 13 | 86 | 0.15 | 8.25 |
| <GO:0009119> | ribonucleoside metabolic p... | BP | 19 | 166 | 0.11 | 8.23 |
| <GO:0055114> | oxidation-reduction proces... | BP | 52 | 841 | 0.06 | 8.16 |
| <GO:0006096> | glycolytic process | BP | 10 | 55 | 0.18 | 8.15 |
| <GO:0046034> | ATP metabolic process | BP | 12 | 77 | 0.16 | 8.08 |
| <GO:1901657> | glycosyl compound metaboli... | BP | 20 | 186 | 0.11 | 8.06 |
| <GO:0009199> | ribonucleoside triphosphat... | BP | 13 | 89 | 0.15 | 8.06 |
| <GO:0090407> | organophosphate biosynthet... | BP | 33 | 421 | 0.08 | 8.06 |
| <GO:0006733> | oxidoreduction coenzyme me... | BP | 11 | 68 | 0.16 | 7.93 |
| <GO:0016051> | carbohydrate biosynthetic ... | BP | 17 | 146 | 0.12 | 7.87 |
| <GO:0051156> | glucose 6-phosphate metabo... | BP | 6 | 23 | 0.26 | 7.87 |
| <GO:0046128> | purine ribonucleoside meta... | BP | 17 | 150 | 0.11 | 7.72 |
| <GO:0016052> | carbohydrate catabolic pro... | BP | 13 | 95 | 0.14 | 7.71 |
| <GO:0042278> | purine nucleoside metaboli... | BP | 17 | 153 | 0.11 | 7.61 |
| <GO:0009259> | ribonucleotide metabolic p... | BP | 26 | 310 | 0.08 | 7.56 |
| <GO:0006163> | purine nucleotide metaboli... | BP | 26 | 311 | 0.08 | 7.54 |
| <GO:0046390> | ribose phosphate biosynthe... | BP | 21 | 220 | 0.10 | 7.54 |
| <GO:0044769> | ATPase activity, coupled t... | MF | 5 | 18 | 0.28 | 7.45 |
| <GO:0009150> | purine ribonucleotide meta... | BP | 25 | 300 | 0.08 | 7.37 |
| <GO:0044724> | single-organism carbohydra... | BP | 12 | 89 | 0.13 | 7.33 |
| <GO:0046496> | nicotinamide nucleotide me... | BP | 9 | 54 | 0.17 | 7.31 |
| <GO:0005975> | carbohydrate metabolic pro... | BP | 37 | 565 | 0.07 | 7.24 |
| <GO:0009260> | ribonucleotide biosyntheti... | BP | 20 | 218 | 0.09 | 7.13 |
| <GO:0019362> | pyridine nucleotide metabo... | BP | 9 | 57 | 0.16 | 7.06 |
| <GO:0032787> | monocarboxylic acid metabo... | BP | 32 | 467 | 0.07 | 7.02 |
| <GO:0043536> | positive regulation of blo... | BP | 5 | 20 | 0.25 | 7.00 |
| <GO:0045333> | cellular respiration | BP | 12 | 96 | 0.12 | 6.95 |
| <GO:1901137> | carbohydrate derivative bi... | BP | 33 | 495 | 0.07 | 6.94 |
| <GO:0015980> | energy derivation by oxida... | BP | 17 | 173 | 0.10 | 6.93 |
| <GO:0009152> | purine ribonucleotide bios... | BP | 19 | 210 | 0.09 | 6.87 |
| <GO:0009165> | nucleotide biosynthetic pr... | BP | 22 | 266 | 0.08 | 6.87 |
| <GO:0019752> | carboxylic acid metabolic ... | BP | 42 | 723 | 0.06 | 6.83 |
| <GO:1901293> | nucleoside phosphate biosy... | BP | 22 | 268 | 0.08 | 6.82 |
| <GO:0016866> | intramolecular transferase... | MF | 5 | 21 | 0.24 | 6.80 |
| <GO:0044283> | small molecule biosyntheti... | BP | 26 | 351 | 0.07 | 6.79 |
| <GO:0072524> | pyridine-containing compou... | BP | 9 | 61 | 0.15 | 6.75 |
| <GO:0006164> | purine nucleotide biosynth... | BP | 19 | 216 | 0.09 | 6.71 |
| <GO:0051188> | cofactor biosynthetic proc... | BP | 12 | 101 | 0.12 | 6.71 |
| <GO:0015078> | hydrogen ion transmembrane... | MF | 10 | 74 | 0.14 | 6.70 |
| <GO:0072521> | purine-containing compound... | BP | 26 | 357 | 0.07 | 6.68 |
| <GO:0044723> | single-organism carbohydra... | BP | 32 | 493 | 0.06 | 6.66 |
| <GO:0022624> | proteasome accessory compl... | CC | 5 | 22 | 0.23 | 6.61 |
| <GO:0006637> | acyl-CoA metabolic process... | BP | 9 | 63 | 0.14 | 6.60 |
| <GO:0035383> | thioester metabolic proces... | BP | 9 | 63 | 0.14 | 6.60 |
| <GO:0046364> | monosaccharide biosyntheti... | BP | 9 | 63 | 0.14 | 6.60 |
| <GO:0006101> | citrate metabolic process | BP | 6 | 31 | 0.19 | 6.56 |
| <GO:0072522> | purine-containing compound... | BP | 19 | 223 | 0.09 | 6.54 |
| <GO:0009108> | coenzyme biosynthetic proc... | BP | 10 | 77 | 0.13 | 6.52 |
| <GO:0006006> | glucose metabolic process | BP | 15 | 153 | 0.10 | 6.50 |
| <GO:0006082> | organic acid metabolic pro... | BP | 43 | 787 | 0.05 | 6.46 |
| <GO:0006094> | gluconeogenesis | BP | 8 | 53 | 0.15 | 6.46 |
| <GO:0043436> | oxoacid metabolic process | BP | 42 | 770 | 0.05 | 6.37 |
| <GO:0022900> | electron transport chain | BP | 7 | 43 | 0.16 | 6.35 |
| <GO:0031970> | organelle envelope lumen | CC | 9 | 67 | 0.13 | 6.33 |
| <GO:0007005> | mitochondrion organization... | BP | 29 | 453 | 0.06 | 6.24 |
| <GO:0019318> | hexose metabolic process | BP | 16 | 179 | 0.09 | 6.23 |
| <GO:0072350> | tricarboxylic acid metabol... | BP | 6 | 34 | 0.18 | 6.19 |
| <GO:0019319> | hexose biosynthetic proces... | BP | 8 | 57 | 0.14 | 6.15 |
| <GO:0015992> | proton transport | BP | 10 | 84 | 0.12 | 6.13 |
| <GO:0008299> | isoprenoid biosynthetic pr... | BP | 5 | 25 | 0.20 | 6.11 |
| <GO:0006818> | hydrogen transport | BP | 10 | 85 | 0.12 | 6.07 |
| <GO:0005996> | monosaccharide metabolic p... | BP | 17 | 204 | 0.08 | 6.06 |
| <GO:0000502> | proteasome complex | CC | 8 | 59 | 0.14 | 6.00 |
| <GO:1902600> | hydrogen ion transmembrane... | BP | 8 | 60 | 0.13 | 5.93 |
| <GO:0006839> | mitochondrial transport | BP | 14 | 155 | 0.09 | 5.88 |
| <GO:1901566> | organonitrogen compound bi... | BP | 30 | 507 | 0.06 | 5.86 |
| <GO:0006099> | tricarboxylic acid cycle | BP | 5 | 27 | 0.19 | 5.83 |
| <GO:0016469> | proton-transporting two-se... | CC | 6 | 38 | 0.16 | 5.76 |
| <GO:0022904> | respiratory electron trans... | BP | 6 | 38 | 0.16 | 5.76 |
| <GO:0006695> | cholesterol biosynthetic p... | BP | 6 | 39 | 0.15 | 5.66 |
| <GO:0016853> | isomerase activity | MF | 12 | 127 | 0.09 | 5.64 |
| <GO:0044255> | cellular lipid metabolic p... | BP | 37 | 727 | 0.05 | 5.50 |
| <GO:0005758> | mitochondrial intermembran... | CC | 7 | 54 | 0.13 | 5.44 |
| <GO:0016126> | sterol biosynthetic proces... | BP | 6 | 42 | 0.14 | 5.39 |
| <GO:0000313> | organellar ribosome | CC | 7 | 55 | 0.13 | 5.37 |
| <GO:0005761> | mitochondrial ribosome | CC | 7 | 55 | 0.13 | 5.37 |
| <GO:0006739> | NADP metabolic process | BP | 5 | 31 | 0.16 | 5.33 |
| <GO:0044262> | cellular carbohydrate meta... | BP | 17 | 239 | 0.07 | 5.27 |
| <GO:0016491> | oxidoreductase activity | MF | 34 | 669 | 0.05 | 5.25 |
| <GO:0016860> | intramolecular oxidoreduct... | MF | 6 | 45 | 0.13 | 5.14 |
| <GO:0006119> | oxidative phosphorylation | BP | 5 | 33 | 0.15 | 5.11 |
| <GO:0008610> | lipid biosynthetic process... | BP | 25 | 441 | 0.06 | 5.09 |
| <GO:0005777> | peroxisome | CC | 11 | 127 | 0.09 | 5.03 |
| <GO:0042579> | microbody | CC | 11 | 127 | 0.09 | 5.03 |
| <GO:0043534> | blood vessel endothelial c... | BP | 7 | 63 | 0.11 | 4.87 |
| <GO:0044712> | single-organism catabolic ... | BP | 35 | 743 | 0.05 | 4.84 |
| <GO:0051896> | regulation of protein kina... | BP | 10 | 115 | 0.09 | 4.81 |
| <GO:0019867> | outer membrane | CC | 11 | 136 | 0.08 | 4.74 |
| <GO:0005741> | mitochondrial outer membra... | CC | 10 | 119 | 0.08 | 4.67 |
| <GO:0006633> | fatty acid biosynthetic pr... | BP | 10 | 119 | 0.08 | 4.67 |
| <GO:0006790> | sulfur compound metabolic ... | BP | 15 | 225 | 0.07 | 4.65 |
| <GO:0010595> | positive regulation of end... | BP | 6 | 52 | 0.12 | 4.64 |
| <GO:0043491> | protein kinase B signaling... | BP | 11 | 140 | 0.08 | 4.63 |
| <GO:0090317> | negative regulation of int... | BP | 7 | 68 | 0.10 | 4.60 |
| <GO:0005840> | ribosome | CC | 12 | 162 | 0.07 | 4.59 |
| <GO:0072330> | monocarboxylic acid biosyn... | BP | 12 | 162 | 0.07 | 4.59 |
| <GO:0000271> | polysaccharide biosyntheti... | BP | 6 | 54 | 0.11 | 4.51 |
| <GO:0009060> | aerobic respiration | BP | 5 | 40 | 0.12 | 4.48 |
| <GO:0043535> | regulation of blood vessel... | BP | 5 | 41 | 0.12 | 4.40 |
| <GO:0051897> | positive regulation of pro... | BP | 7 | 72 | 0.10 | 4.40 |


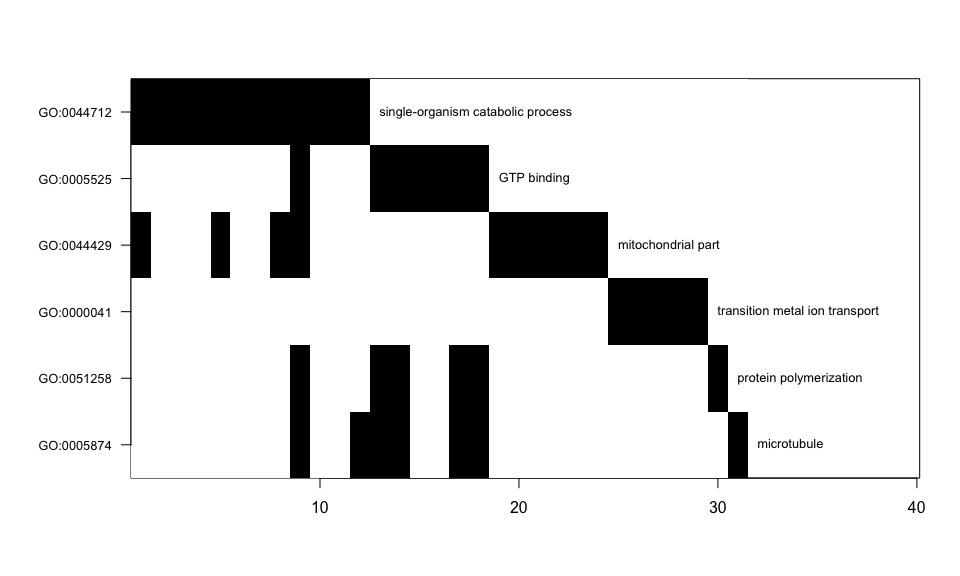


Table SS-6: Gene Ontology terms enriched for multi-t-test CR genes, heart; set.mean is proportion of GO term that is CR-related

|  | Term | Ontology | CRgenes | set.size | set.mean | z.score |
| --- | --- | --- | --- | --- | --- | --- |
| <GO:0051258> | protein polymerization | BP | 6 | 173 | 0.03 | 5.91 |
| <GO:0003924> | GTPase activity | MF | 6 | 192 | 0.03 | 5.52 |
| <GO:0005525> | GTP binding | MF | 7 | 299 | 0.02 | 4.88 |
| <GO:0005874> | microtubule | CC | 7 | 304 | 0.02 | 4.82 |
| <GO:0044712> | single-organism catabolic ... | BP | 12 | 743 | 0.02 | 4.79 |
| <GO:0032561> | guanyl ribonucleotide bind... | MF | 7 | 318 | 0.02 | 4.66 |
| <GO:0019001> | guanyl nucleotide binding | MF | 7 | 319 | 0.02 | 4.65 |
| <GO:0044429> | mitochondrial part | CC | 10 | 592 | 0.02 | 4.53 |


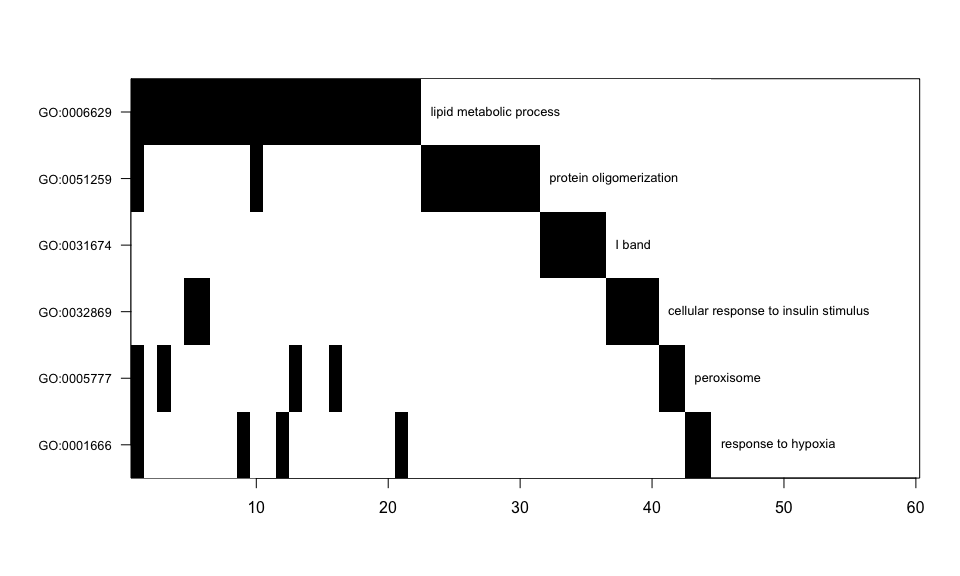


Table SS-7: Gene Ontology terms enriched for multi-t-test CR genes, gastrocnemius; set.mean is proportion of GO term that is CR related

|  | Term | Ontology | CRgenes | set.size | set.mean | z.score |
| --- | --- | --- | --- | --- | --- | --- |
| <GO:0006639> | acylglycerol metabolic pro... | BP | 7 | 89 | 0.08 | 7.74 |
| <GO:0006638> | neutral lipid metabolic pr... | BP | 7 | 91 | 0.08 | 7.64 |
| <GO:0006641> | triglyceride metabolic pro... | BP | 6 | 77 | 0.08 | 7.12 |
| <GO:0046486> | glycerolipid metabolic pro... | BP | 10 | 231 | 0.04 | 6.29 |
| <GO:0044255> | cellular lipid metabolic p... | BP | 18 | 727 | 0.02 | 5.44 |
| <GO:0006629> | lipid metabolic process | BP | 22 | 992 | 0.02 | 5.44 |
| <GO:0005777> | peroxisome | CC | 6 | 127 | 0.05 | 5.16 |
| <GO:0042579> | microbody | CC | 6 | 127 | 0.05 | 5.16 |
| <GO:0032869> | cellular response to insul... | BP | 6 | 132 | 0.05 | 5.03 |
| <GO:0031674> | I band | CC | 5 | 101 | 0.05 | 4.87 |
| <GO:0008610> | lipid biosynthetic process... | BP | 12 | 441 | 0.03 | 4.80 |
| <GO:0030258> | lipid modification | BP | 6 | 148 | 0.04 | 4.64 |
| <GO:0045834> | positive regulation of lip... | BP | 5 | 111 | 0.05 | 4.56 |
| <GO:0051259> | protein oligomerization | BP | 11 | 418 | 0.03 | 4.46 |
| <GO:0001666> | response to hypoxia | BP | 6 | 156 | 0.04 | 4.46 |
| <GO:0036293> | response to decreased oxyg... | BP | 6 | 159 | 0.04 | 4.40 |
| <GO:0032868> | response to insulin | BP | 6 | 160 | 0.04 | 4.38 |
|  |  |  |  |  |  |  |


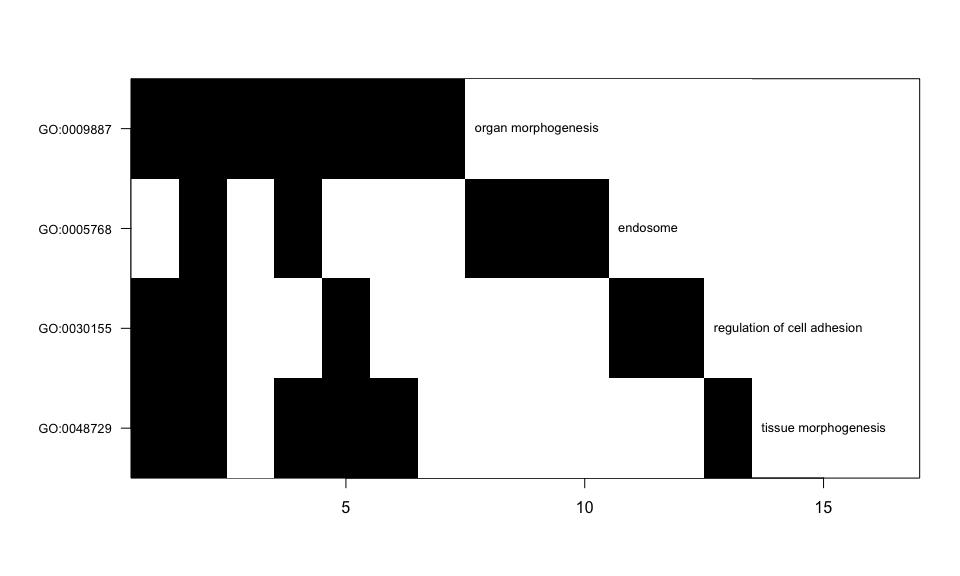


Table SS-8: Gene Ontology terms enriched for multi-t-test CR genes, cortex; set.mean is proportion of GO term that is CR related

|  | Term | Ontology | CRgenes | set.size | set.mean | z.score |
| --- | --- | --- | --- | --- | --- | --- |
| <GO:0048729> | tissue morphogenesis | BP | 6 | 582 | 0.01 | 5.67 |
| <GO:0009887> | organ morphogenesis | BP | 7 | 861 | 0.01 | 5.24 |
| <GO:0030155> | regulation of cell adhesio... | BP | 5 | 506 | 0.01 | 5.02 |
| <GO:0030334> | regulation of cell migrati... | BP | 5 | 543 | 0.01 | 4.79 |
| <GO:2000145> | regulation of cell motilit... | BP | 5 | 571 | 0.01 | 4.63 |
| <GO:0040007> | growth | BP | 6 | 832 | 0.01 | 4.44 |
| <GO:0005768> | endosome | CC | 5 | 608 | 0.01 | 4.43 |

### References

- De Magalhães JP, Curado J, Church GM. Meta-analysis of age-related gene expression profiles identifies common signatures of aging. Bioinformatics. 2009;25(7):875-881. <doi:10.1093/bioinformatics/btp073>.
- Hao, L, He, Q, Wang, Z, Craven, M, Newton, MA, and Ahlquist, P (2013). Limited agreement of independent RNAi screens for virus-required host genes owes more to false-negative than false-positive factors. PLoS Comput Biol 9(9): e1003235.
- Newton, M.A., Quintana, F.A., den Boon, J.A., Sengupta, S. and Ahlquist, P. (2007). Random-set methods identify distinct aspects of the enrichment signal in gene-set analysis. Annals of Applied Statistics , 1, 85-106.
- Storey, John D. The positive false discovery rate: a Bayesian interpretation and the q -value. Ann. Statist. 31 (2003), no. 6, 2013--2035. <doi:10.1214/aos/1074290335>. <http://projecteuclid.org/euclid.aos/1074290335>.
- Thomas, Marlin A., and Audrey E. Taub. "Calculating binomial probabilities when the trial probabilities are unequal." Journal of Statistical Computation and Simulation 14.2 (1982): 125-131.

1. Technical Report no. 47, University of Wisconsin Stochastic Modeling Lab. Research supported in part by U54AI117924. [↑](#footnote-ref-1)
